# Supplementary figures and images for: eXplainable Artificial Intelligence (XAI) for the identification of biologically relevant gene expression patterns in longitudinal human studies, insights from obesity research
Source: PLoS Comput Biol. 2020 Apr 10;16(4):e1007792. doi: 10.1371/journal.pcbi.1007792 (PMC7176286; doi:10.1371/journal.pcbi.1007792)

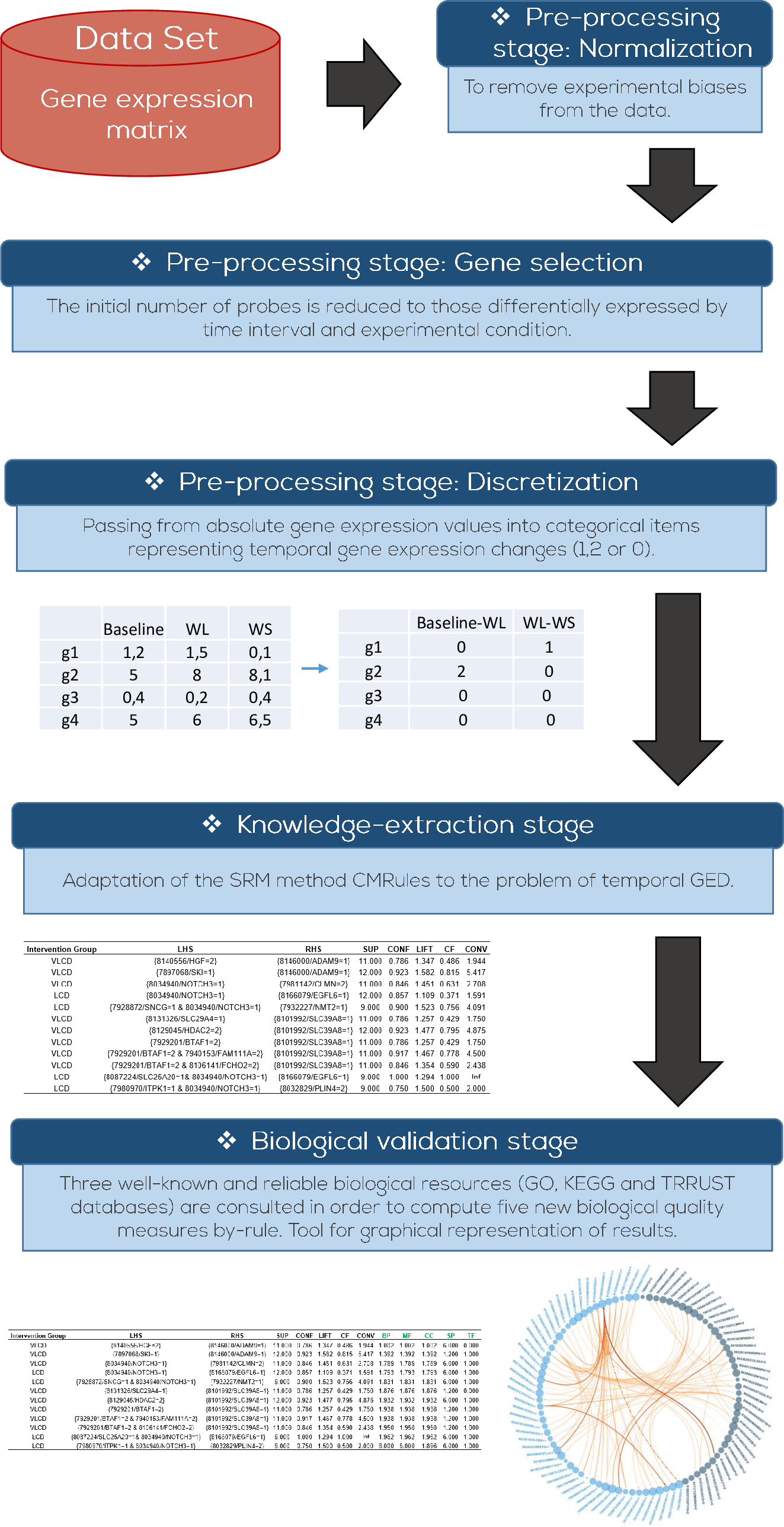

Supplement: S1 Fig — The values (0,1 and 2) from the discretization step correspond to (‘No change in gene expression’,’Downregulation’ and ‘Upregulation). In order to simplify the figure, the data from the example only represent gene expression values from one individual in the population. Thus, and since our discretization approach uses the mean SLR (from all individuals in a dataset) for computing discrete states, they should not be intended as an example of a real discretization process in our approach. (TIF) [file pcbi.1007792.s001.tif]

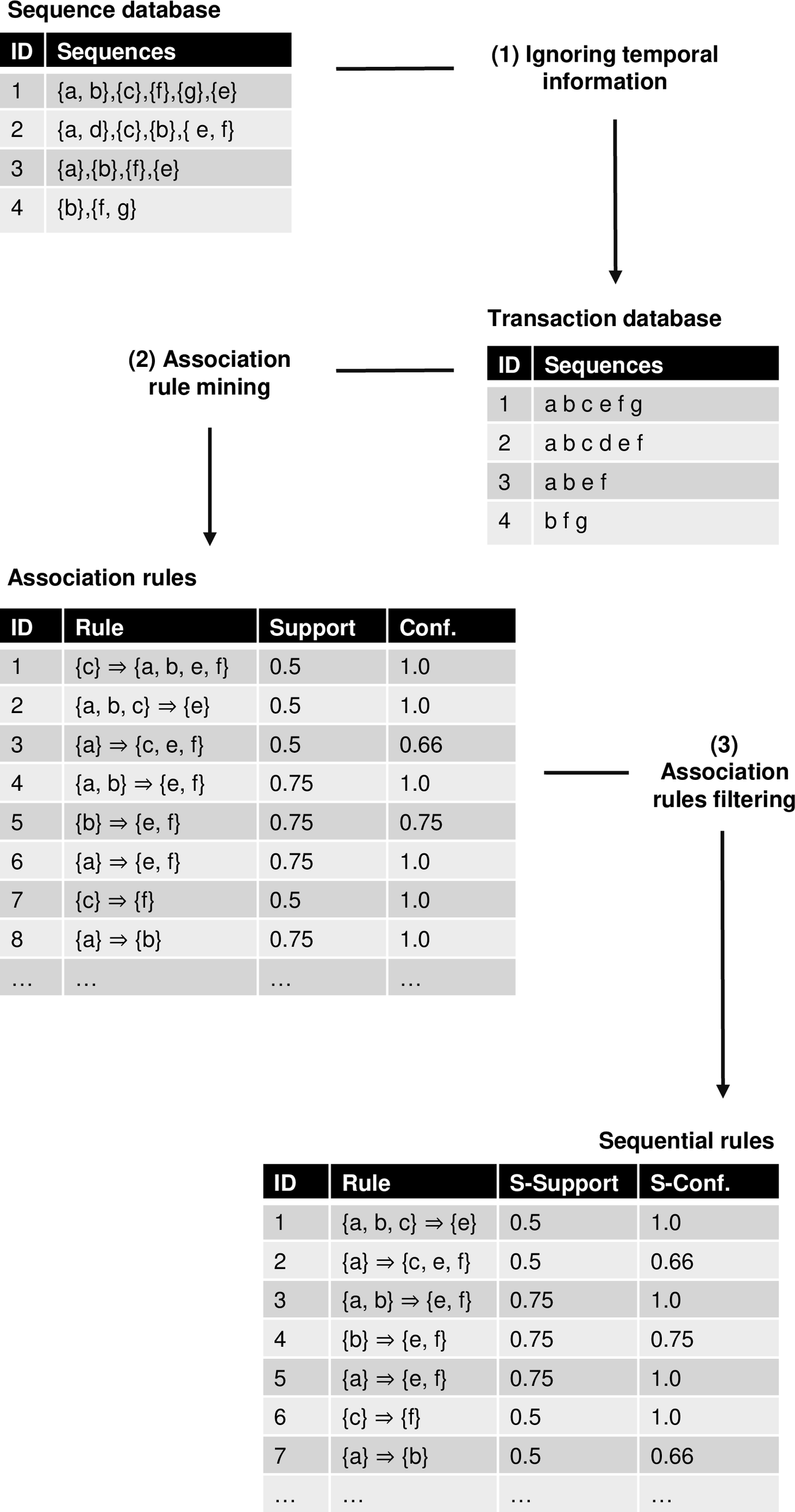

Supplement: S2 Fig — This figure is a modification of the Fig 3 available in the original publication of CMRules [25]. (TIF) [file pcbi.1007792.s002.tif]

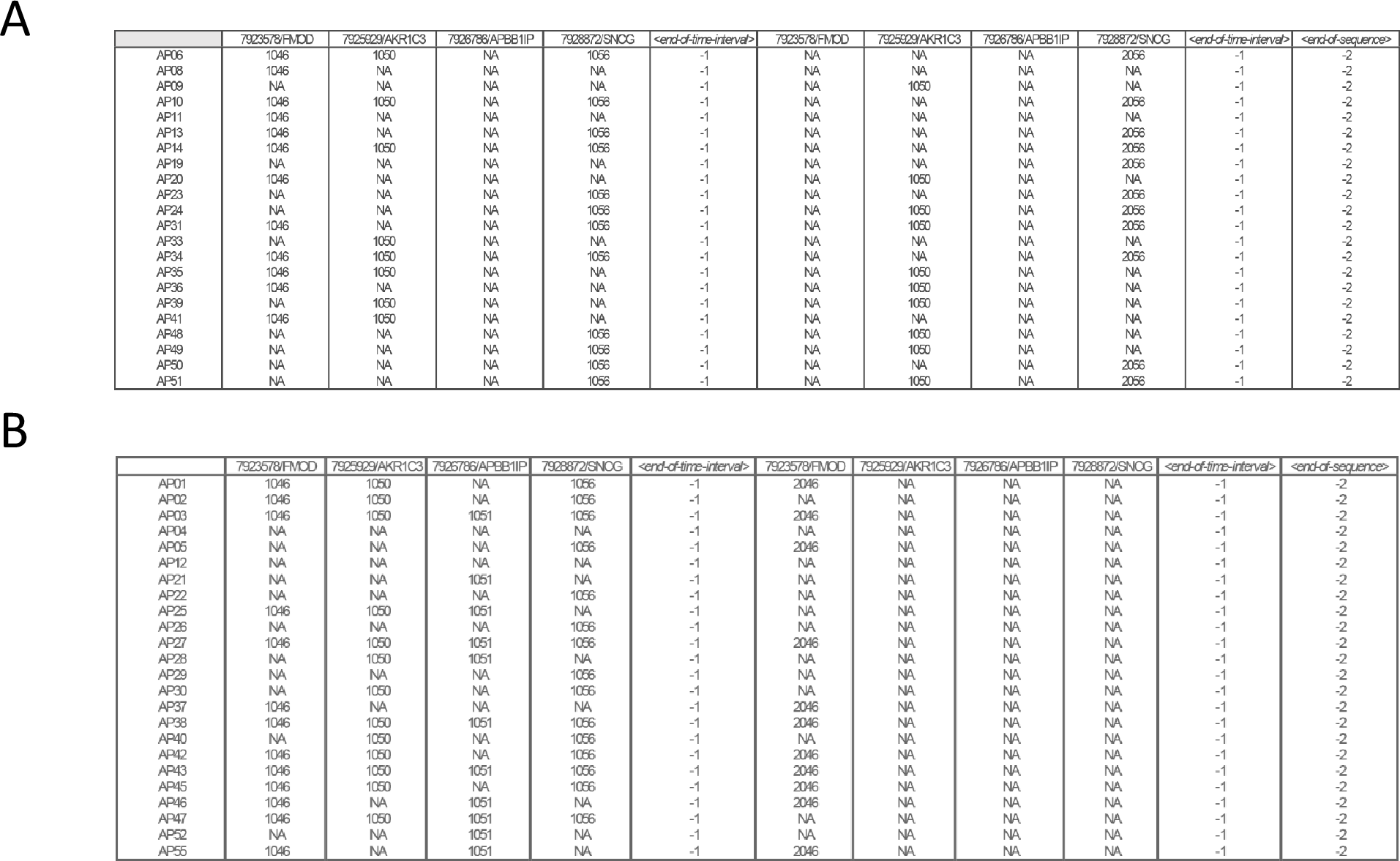

Supplement: S3 Fig — Each database must contain discrete temporal information (events) for changes in gene expression. Events were represented using a 4-digit code (where the first digit represents the change in gene expression (1 = Downregulation, 2 = Upregulation, NA = no change) and the next three digits represent the identifier for the i probe under study). Section A refers to the sequence database constructed in the GSE77962 dataset (VLCD group) and B to the sequence database constructed in the GSE77962 dataset (LCD group). (TIF) [file pcbi.1007792.s003.tif]

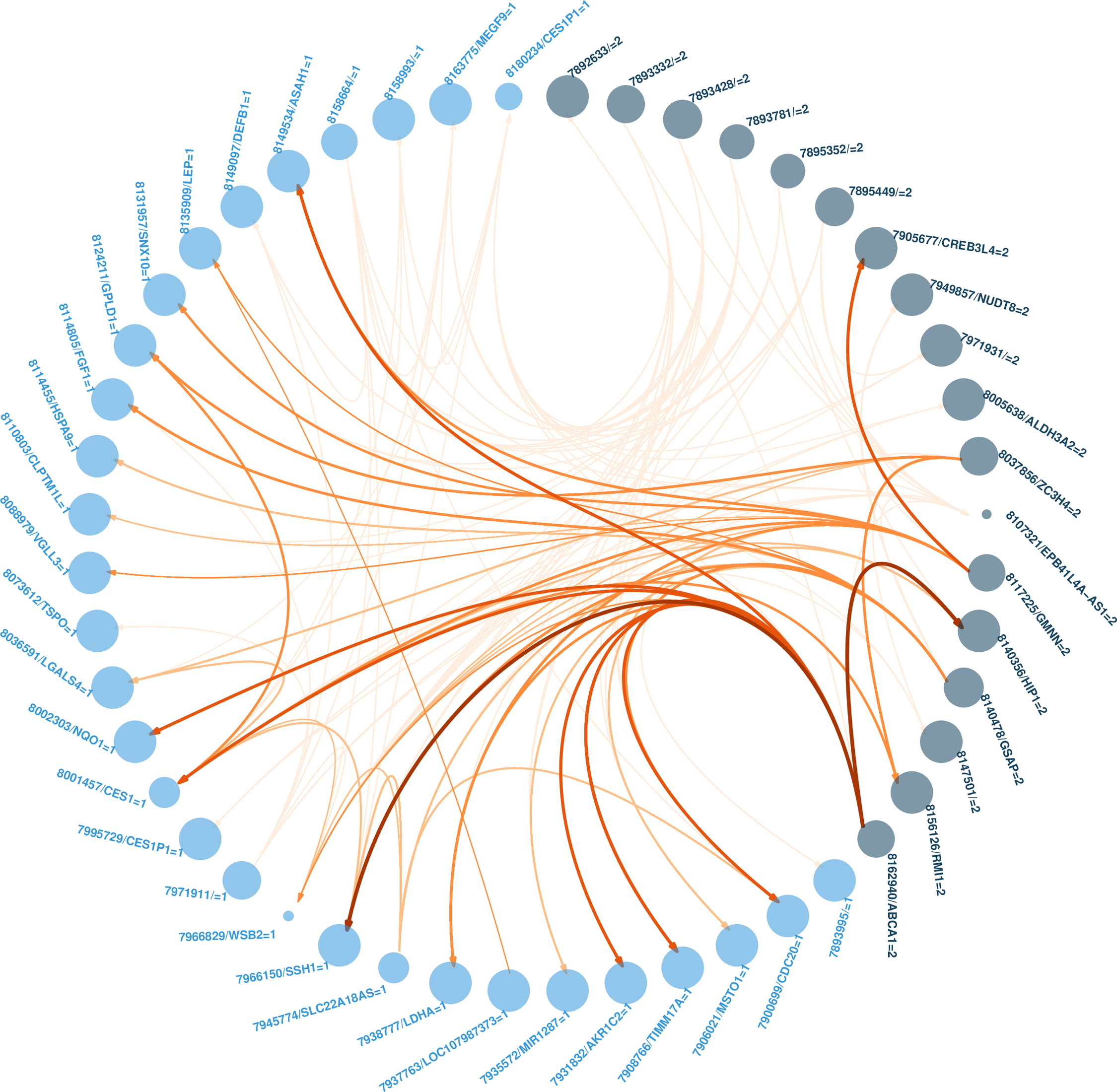

Supplement: S5 Fig — (TIF) [file pcbi.1007792.s005.tif]

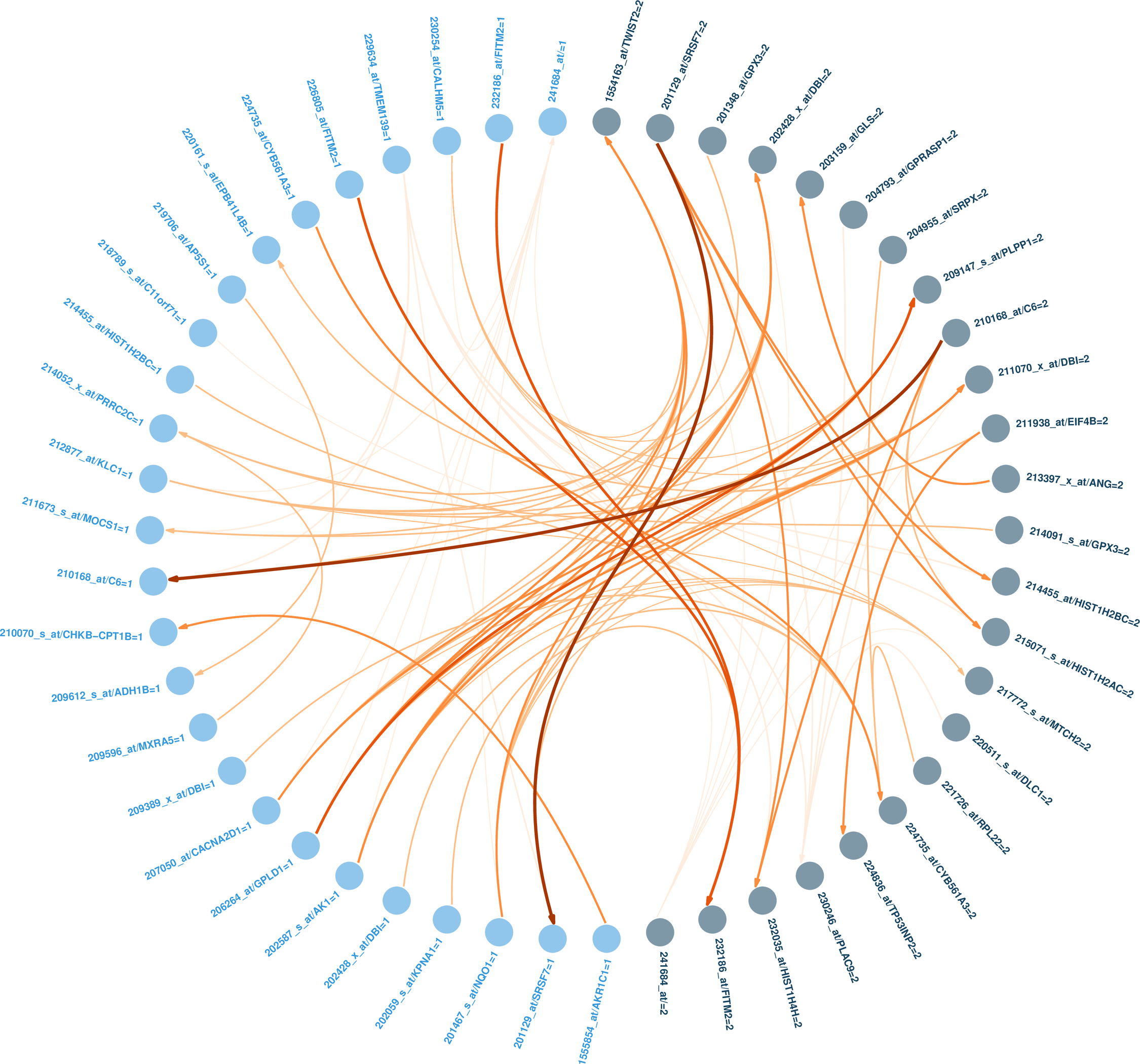

Supplement: S6 Fig — (TIF) [file pcbi.1007792.s006.tif]

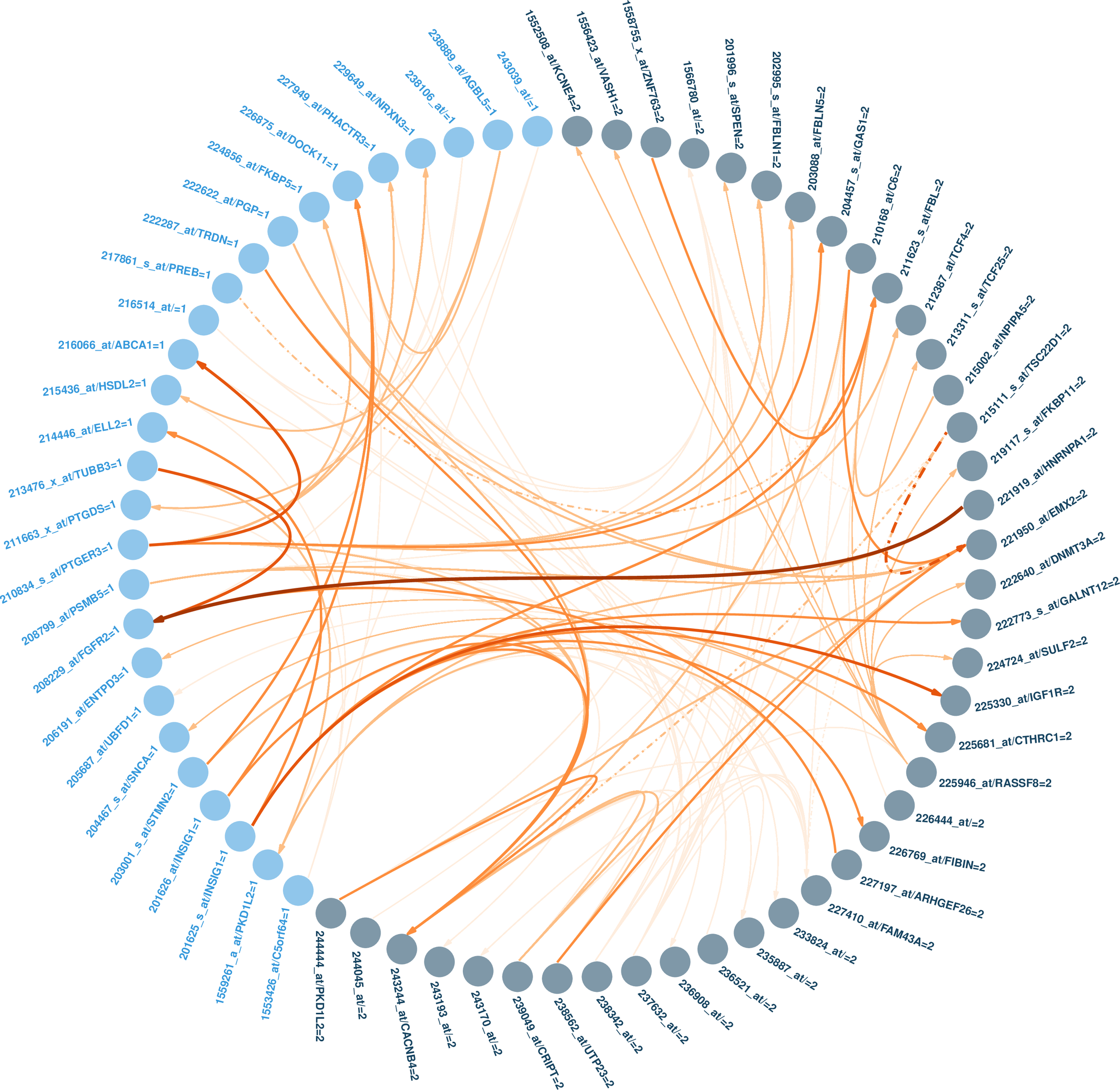

Supplement: S7 Fig — (TIF) [file pcbi.1007792.s007.tif]

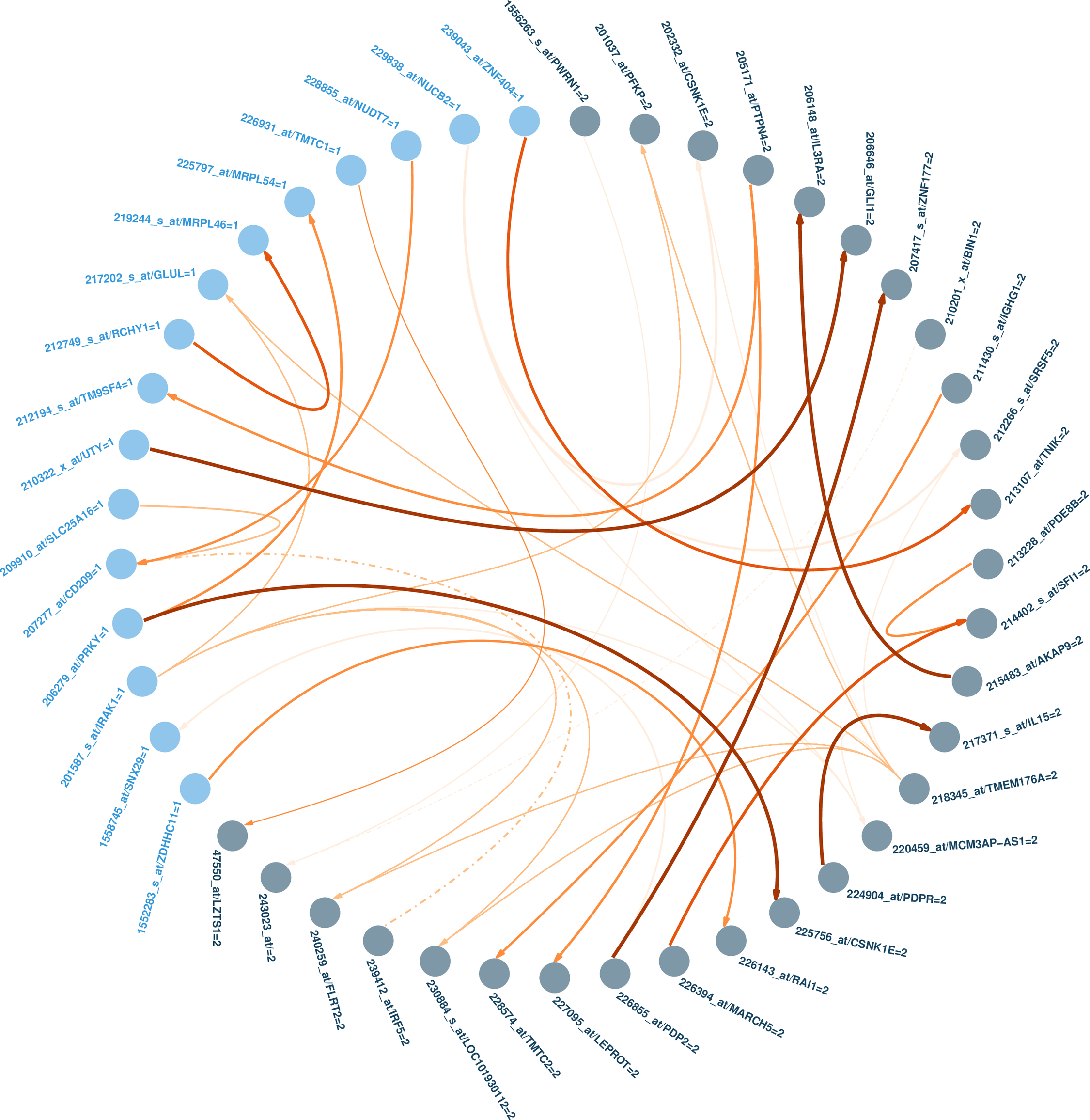

Supplement: S8 Fig — (TIF) [file pcbi.1007792.s008.tif]

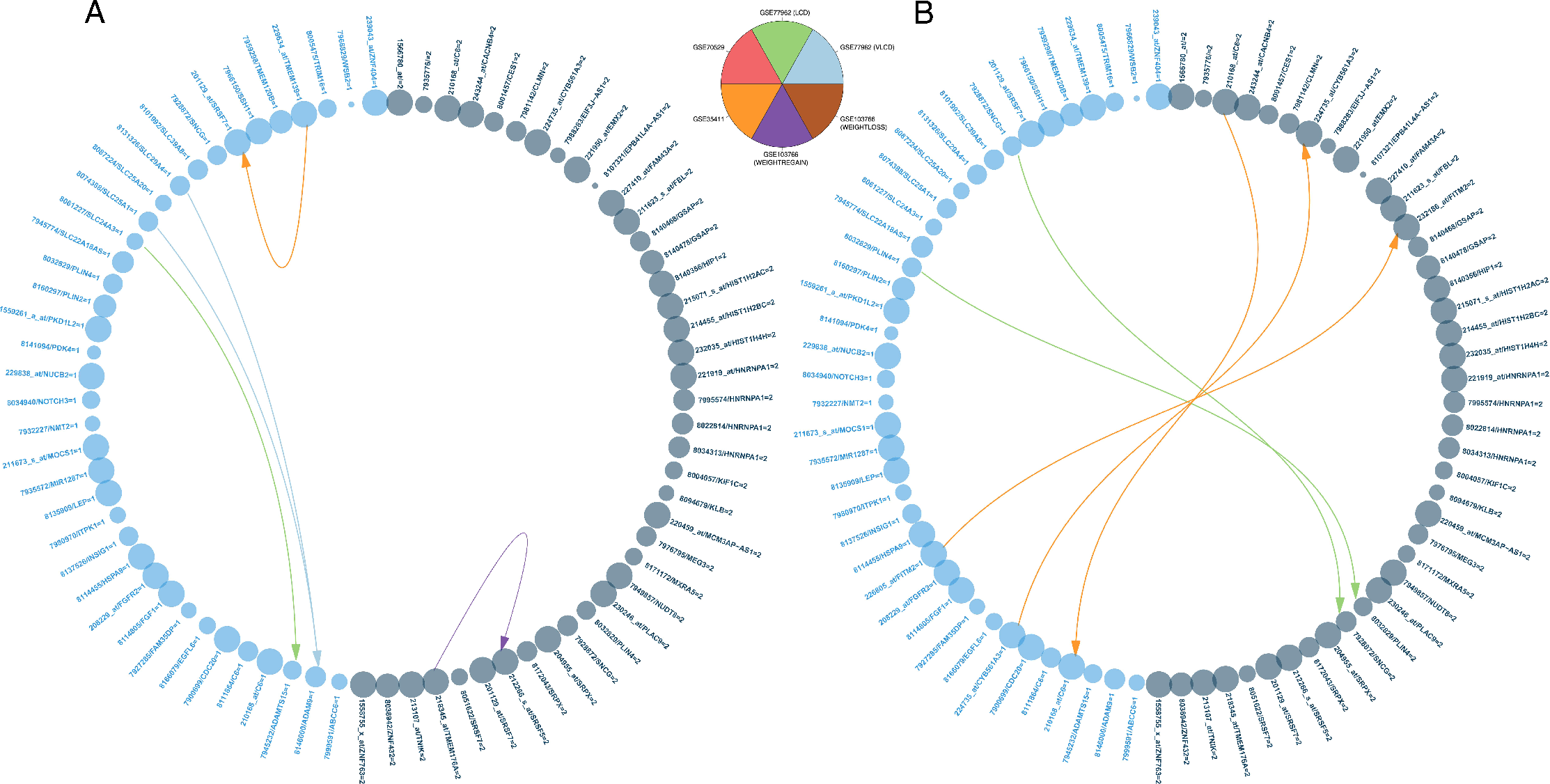

Supplement: S9 Fig — The complete set of sequential rules that contain at least one of the identified key LHS and RHS loci (from the validation study) is available in the S3 Table. A refers to validated patterns between datasets while B illustrates rebound effects in the gene expression of certain loci during the dietary intervention program evidenced in different datasets. (TIF) [file pcbi.1007792.s009.tif]
