# Supplementary material for: eXplainable Artificial Intelligence (XAI) for the identification of biologically relevant gene expression patterns in longitudinal human studies, insights from obesity research
Source: PLoS Comput Biol. 2020 Apr 10;16(4):e1007792. doi: 10.1371/journal.pcbi.1007792 (PMC7176286; doi:10.1371/journal.pcbi.1007792)
Supplement: S1 Table — (PDF) [file pcbi.1007792.s010.pdf]

Supplementary table 1. 50 output sequential rules identified from the discovery GSE77962 dataset (LCD group).

LHS	RHS	BP	CC	CF	CONF	CONV	LIFT	MF	SP	SUP	TF
{7916432/DHCR24=1}	{7932227/NMT2=1}	1.61	1.61	0.59	0.83	2.45	1.41	1.61	6.00	10.00	0.00
{7916432/DHCR24=1}	{8166079/EGFL6=1}	1.84	1.84	0.63	0.92	2.73	1.19	1.84	6.00	11.00	0.00
{7923578/FMOD=1}	{8029530/APOE=2}	1.38	1.38	0.45	0.75	1.82	1.38	6.00	6.00	9.00	0.00
{7928872/SNCG=1}	{7932227/NMT2=1}	1.78	1.78	0.39	0.75	1.64	1.27	1.78	1.20	9.00	0.00
{7928872/SNCG=1}	{8166079/EGFL6=1}	1.87	1.87	0.27	0.83	1.36	1.08	1.87	1.20	10.00	0.00
{7928872/SNCG=1}	{7928872/SNCG=2}	1.00	1.00	0.67	0.83	3.00	1.67	1.00	1.20	10.00	0.00
{7974341/GNG2=1}	{7930921/BAG3=1}	1.74	1.74	0.32	0.69	1.48	1.27	1.74	6.00	9.00	0.00
{8166079/EGFL6=1}	{7930921/BAG3=1}	1.84	1.84	0.09	0.59	1.10	1.08	1.84	1.20	10.00	0.00
{7948612/FADS1=1}	{7932227/NMT2=1}	1.64	1.64	0.56	0.82	2.25	1.38	1.64	6.00	9.00	0.00
{7974341/GNG2=1}	{7932227/NMT2=1}	1.77	1.77	0.62	0.85	2.66	1.43	1.77	6.00	11.00	0.00
{7989670/RBPMS2=1}	{7932227/NMT2=1}	1.69	1.69	0.39	0.75	1.64	1.27	1.69	1.20	9.00	0.00
{8002303/NQO1=1}	{7932227/NMT2=1}	1.71	1.71	0.19	0.67	1.23	1.13	1.71	1.20	12.00	0.00
{8034940/NOTCH3=1}	{7932227/NMT2=1}	1.59	1.59	0.13	0.64	1.15	1.09	1.59	6.00	9.00	1.00
{8061227/SLC24A3=1}	{7932227/NMT2=1}	1.87	1.87	0.13	0.64	1.15	1.09	1.87	1.20	9.00	0.00
{8061227/SLC24A3=1}	{7945232/ADAMTS15=1}	1.92	1.92	0.29	0.64	1.40	1.29	1.92	1.20	9.00	0.00
{8172043/SRPX=2}	{7945232/ADAMTS15=1}	1.94	1.94	0.50	0.75	2.00	1.50	6.00	1.20	9.00	0.00
{7974341/GNG2=1}	{8005475/TRIM16|TRIM16L=1}	1.67	1.67	0.66	0.92	2.95	1.19	1.67	6.00	12.00	0.00
{7974341/GNG2=1}	{8124211/GPLD1=1}	1.48	1.48	0.38	0.69	1.63	1.38	1.48	6.00	9.00	0.00
{7974341/GNG2=1}	{8137526/INSIG1=1}	1.74	1.74	0.38	0.69	1.63	1.38	1.74	6.00	9.00	0.00
{7974341/GNG2=1}	{8029530/APOE=2}	1.44	1.44	0.32	0.69	1.48	1.27	1.44	6.00	9.00	0.00
{7980970/ITPK1=1}	{8032829/PLIN4=2}	6.00	1.90	0.50	0.75	2.00	1.50	6.00	6.00	9.00	0.00

{7995729/CES1P1=1}	{8166079/EGFL6=1}	6.00	6.00	-0.21	0.61	0.58	0.79	6.00	1.20	11.00	0.00
{8002303/NQO1=1}	{8166079/EGFL6=1}	1.88	1.88	0.27	0.83	1.36	1.08	1.88	1.20	15.00	0.00
{8061227/SLC24A3=1}	{8005475/TRIM16|TRIM16L=1}	1.84	1.84	0.37	0.86	1.59	1.11	1.84	1.20	12.00	0.00
{8022747/B4GALT6=1}	{8029530/APOE=2}	1.50	1.50	0.60	0.82	2.50	1.50	1.50	6.00	9.00	0.00
{8032829/PLIN4=1}	{8111864/C6=1}	6.00	1.92	0.48	0.69	1.92	1.69	6.00	6.00	9.00	0.00
{8032829/PLIN4=1}	{7928872/SNCG=2}	6.00	1.89	0.38	0.69	1.63	1.38	6.00	1.20	9.00	0.00
{8032829/PLIN4=1}	{8001457/CES1=2}	6.00	1.89	0.44	0.69	1.77	1.52	6.00	6.00	9.00	0.00
{8032829/PLIN4=1}	{8032829/PLIN4=2}	6.00	1.83	0.54	0.77	2.17	1.54	6.00	1.20	10.00	0.00
{8034940/NOTCH3=1}	{8166079/EGFL6=1}	1.79	1.79	0.37	0.86	1.59	1.11	1.79	6.00	12.00	1.00
{8034940/NOTCH3=1}	{8004057/KIF1C=2}	1.76	1.76	0.35	0.64	1.53	1.41	1.76	6.00	9.00	1.00
{8034940/NOTCH3=1}	{8032829/PLIN4=2}	6.00	1.84	0.29	0.64	1.40	1.29	6.00	6.00	9.00	1.00
{8061227/SLC24A3=1}	{8137526/INSIG1=1}	1.86	1.86	0.43	0.71	1.75	1.43	1.86	1.20	10.00	0.00
{8061227/SLC24A3=1}	{7988283/EIF3J-AS1=2}	6.00	6.00	0.21	0.64	1.27	1.18	6.00	1.20	9.00	0.00
{8061227/SLC24A3=1}	{8032829/PLIN4=2}	6.00	1.94	0.29	0.64	1.40	1.29	6.00	1.20	9.00	0.00
{8082965/MRAS=1}	{8166079/EGFL6=1}	1.82	1.82	0.60	0.91	2.50	1.18	1.82	6.00	10.00	0.00
{8087224/SLC25A20=1}	{8166079/EGFL6=1}	1.96	1.96	0.27	0.83	1.36	1.08	1.96	1.20	10.00	0.00
{7974341/GNG2=1,7916432/DHCR24=1}	{7932227/NMT2=1}	1.81	1.81	0.76	0.90	4.09	1.52	1.81	6.00	9.00	0.00
{7916432/DHCR24=1,8002303/NQO1=1}	{7932227/NMT2=1}	1.74	1.74	0.56	0.82	2.25	1.38	1.74	6.00	9.00	0.00
{7916432/DHCR24=1,8002303/NQO1=1}	{8166079/EGFL6=1}	1.92	1.92	1.00	1.00	Inf	1.29	1.92	6.00	11.00	0.00
{8022747/B4GALT6=1,7923578/FMOD=1}	{8029530/APOE=2}	1.71	1.71	0.60	0.82	2.50	1.50	6.00	6.00	9.00	0.00
{7928872/SNCG=1,8034940/NOTCH3=1}	{7932227/NMT2=1}	1.83	1.83	0.76	0.90	4.09	1.52	1.83	6.00	9.00	1.00
{7989670/RBPMS2=1,7974341/GNG2=1}	{7932227/NMT2=1}	1.85	1.85	0.76	0.90	4.09	1.52	1.85	6.00	9.00	0.00

{7974341/GNG2=1,8002303/NQO1=1}	{7932227/NMT2=1}	1.84	1.84	0.78	0.91	4.50	1.54	1.84	6.00	10.00	0.00
{8061227/SLC24A3=1,7974341/GNG2=1}	{7932227/NMT2=1}	1.90	1.90	0.56	0.82	2.25	1.38	1.90	6.00	9.00	0.00
{7989670/RBPMS2=1,8061227/SLC24A3=1}	{7932227/NMT2=1}	1.93	1.93	0.56	0.82	2.25	1.38	1.93	1.20	9.00	0.00
{8061227/SLC24A3=1,7974341/GNG2=1}	{8137526/INSIG1=1}	1.91	1.91	0.64	0.82	2.75	1.64	1.91	6.00	9.00	0.00
{7980970/ITPK1=1,8034940/NOTCH3=1}	{8032829/PLIN4=2}	6.00	1.90	0.50	0.75	2.00	1.50	6.00	6.00	9.00	1.00
{8087224/SLC25A20=1,8034940/NOTCH3=1}	{8166079/EGFL6=1}	1.96	1.96	1.00	1.00	Inf	1.29	1.96	6.00	9.00	1.00
{7989670/RBPMS2=1,8061227/SLC24A3=1,7974341/GNG2=1}	{7932227/NMT2=1}	1.93	1.93	0.76	0.90	4.09	1.52	1.93	6.00	9.00	0.00
