# Supplementary material for: eXplainable Artificial Intelligence (XAI) for the identification of biologically relevant gene expression patterns in longitudinal human studies, insights from obesity research
Source: PLoS Comput Biol. 2020 Apr 10;16(4):e1007792. doi: 10.1371/journal.pcbi.1007792 (PMC7176286; doi:10.1371/journal.pcbi.1007792)
Supplement: S2 Table — (PDF) [file pcbi.1007792.s011.pdf]

Supplementary table 2. 50 output sequential rules identified from the discovery GSE77962 dataset (VLCD group).

LHS	RHS	BP	CC	CF	CONF	CONV	LIFT	MF	SP	SUP	TF
{7897068/SKI=1}	{8146000/ADAM9=1}	1.39	1.39	0.82	0.92	5.42	1.58	1.39	1.20	12.00	1.00
{7897068/SKI=1}	{7981142/CLMN=2}	1.79	1.79	0.63	0.85	2.71	1.45	1.79	1.20	11.00	1.00
{7899173/DHDDS=1}	{7948344/GLYAT=2}	1.83	1.83	0.47	0.73	1.88	1.47	1.83	6.00	11.00	0.00
{7899173/DHDDS=1}	{8094679/KLB=2}	1.76	1.76	0.42	0.73	1.72	1.35	1.76	6.00	11.00	0.00
{8042942/HK2=1}	{7907160/ATP1B1=1}	1.42	1.42	0.60	0.79	2.53	1.71	1.42	6.00	11.00	0.00
{7912056/PLEKHG5|TNFRSF25=1}	{8180268/CYP51A1=1}	1.87	1.87	0.82	0.92	5.50	1.69	1.87	6.00	11.00	0.00
{7912692/HSPB7=1}	{8141094/PDK4=1}	1.83	1.83	0.63	0.85	2.71	1.45	1.83	1.20	11.00	0.00
{7912692/HSPB7=1}	{8146000/ADAM9=1}	1.89	1.89	0.63	0.85	2.71	1.45	1.89	1.20	11.00	0.00
{7912692/HSPB7=1}	{7981142/CLMN=2}	1.91	1.91	0.63	0.85	2.71	1.45	1.91	1.20	11.00	0.00
{8053484/ST3GAL5=2}	{7914342/FABP3=1}	1.84	1.84	0.71	0.86	3.50	1.71	1.84	6.00	12.00	0.00
{8053484/ST3GAL5=2}	{7917304/MCOLN3=1}	1.84	1.84	0.53	0.79	2.14	1.45	1.84	6.00	11.00	0.00
{7980970/ITPK1=1}	{7927285/FAM35DP|RHEB=1}	1.70	1.70	0.23	0.65	1.30	1.19	1.70	6.00	11.00	0.00
{8180257/CEP170=2}	{7927710/CDK1=1}	6.00	1.78	0.60	0.79	2.53	1.71	1.78	6.00	11.00	0.00
{7928872/SNCG=1}	{8101992/SLC39A8=1}	1.90	1.90	0.47	0.80	1.88	1.28	1.90	1.20	12.00	0.00
{7928872/SNCG=1}	{8146000/ADAM9=1}	1.71	1.71	0.36	0.73	1.56	1.26	1.71	1.20	11.00	0.00
{7936322/GPAM=1}	{8160297/PLIN2=1}	1.79	1.79	0.69	0.85	3.25	1.69	6.00	6.00	11.00	0.00
{7936322/GPAM=1}	{8019392/FASN=2}	1.49	1.49	0.69	0.85	3.25	1.69	1.49	6.00	11.00	0.00

{7948344/GLYAT=1}	{7948344/GLYAT=2}	1.58	1.58	0.83	0.92	6.00	1.83	1.58	1.20	11.00	0.00
{7959298/TMEM120B=1}	{8146000/ADAM9=1}	1.88	1.88	0.82	0.92	5.42	1.58	1.88	1.20	12.00	0.00
{7959298/TMEM120B=1}	{8160297/PLIN2=1}	1.92	1.92	0.69	0.85	3.25	1.69	6.00	1.20	11.00	0.00
{7959298/TMEM120B=1}	{7981142/CLMN=2}	1.88	1.88	0.63	0.85	2.71	1.45	1.88	1.20	11.00	0.00
{7963427/KRT5=1}	{8138466/MACC1=1}	1.89	1.89	0.66	0.85	2.98	1.56	1.89	1.20	11.00	0.00
{7963427/KRT5=1}	{8141094/PDK4=1}	1.86	1.86	0.63	0.85	2.71	1.45	1.86	1.20	11.00	0.00
{7963880/ITGA7=1}	{8146000/ADAM9=1}	1.68	1.68	0.63	0.85	2.71	1.45	1.68	6.00	11.00	0.00
{7980970/ITPK1=1}	{8101992/SLC39A8=1}	1.92	1.92	0.22	0.71	1.28	1.13	1.92	6.00	12.00	0.00
{7980970/ITPK1=1}	{8141094/PDK4=1}	1.69	1.69	0.15	0.65	1.18	1.11	1.69	6.00	11.00	0.00
{7980970/ITPK1=1}	{7935776/=2}	6.00	6.00	0.29	0.65	1.42	1.29	6.00	6.00	11.00	0.00
{7980970/ITPK1=1}	{8094679/KLB=2}	1.80	1.80	0.23	0.65	1.30	1.19	1.80	6.00	11.00	0.00
{7980970/ITPK1=1}	{8171172/MXRA5=2}	1.98	1.98	0.15	0.65	1.18	1.11	1.98	6.00	11.00	0.00
{7991138/NMB=1}	{8146000/ADAM9=1}	1.75	1.75	0.49	0.79	1.94	1.35	1.75	1.20	11.00	0.00
{7991846/DECR2|NME4=1}	{8101992/SLC39A8=1}	1.91	1.91	0.59	0.85	2.44	1.35	1.91	6.00	11.00	0.00
{7999591/ABCC6|ABCC6P1|ABCC6P2|LOC105369239=1}	{7981142/CLMN=2}	1.91	1.91	0.25	0.69	1.33	1.18	1.91	1.20	11.00	0.00
{8011188/TLCD2=1}	{8141094/PDK4=1}	6.00	1.99	0.63	0.85	2.71	1.45	6.00	1.20	11.00	0.00
{8011188/TLCD2=1}	{8180268/CYP51A1=1}	6.00	1.97	0.66	0.85	2.98	1.56	6.00	6.00	11.00	0.00
{8011188/TLCD2=1}	{7961673/GYS2=2}	6.00	1.99	0.66	0.85	2.98	1.56	6.00	6.00	11.00	0.00
{8016033/FAM171A2=1}	{8101992/SLC39A8=1}	6.00	1.97	0.29	0.73	1.41	1.17	6.00	1.20	11.00	0.00

{8016832/MMD=1}	{7948344/GLYAT=2}	1.84	1.84	0.47	0.73	1.88	1.47	1.84	1.20	11.00	0.00
{8023995/FSTL3=1}	{8146000/ADAM9=1}	1.49	1.49	0.36	0.73	1.56	1.26	1.49	1.20	11.00	0.00
{8023995/FSTL3=1}	{7948344/GLYAT=2}	1.78	1.78	0.47	0.73	1.88	1.47	1.78	1.20	11.00	0.00
{8024323/REEP6=1}	{8101992/SLC39A8=1}	1.90	1.90	0.47	0.80	1.88	1.28	1.90	1.20	12.00	0.00
{8024323/REEP6=1}	{8141094/PDK4=1}	1.86	1.86	0.36	0.73	1.56	1.26	1.86	1.20	11.00	0.00
{8024323/REEP6=1}	{8094679/KLB=2}	1.91	1.91	0.42	0.73	1.72	1.35	1.91	1.20	11.00	0.00
{8033818/OLFM2=1}	{8033825/COL5A3=2}	1.84	1.84	0.42	0.73	1.72	1.35	1.84	6.00	11.00	0.00
{8034544/PRDX2=1}	{8101992/SLC39A8=1}	1.93	1.93	0.33	0.75	1.50	1.20	1.93	1.20	12.00	0.00
{8034544/PRDX2=1}	{8146000/ADAM9=1}	1.78	1.78	0.25	0.69	1.33	1.18	1.78	1.20	11.00	0.00
{8034544/PRDX2=1}	{8121405/C6orf183=2}	6.00	6.00	0.25	0.69	1.33	1.18	6.00	1.20	11.00	0.00
{8034783/ADGRL1=1}	{8141094/PDK4=1}	1.87	1.87	0.15	0.65	1.18	1.11	1.87	1.20	11.00	0.00
{8034783/ADGRL1=1}	{7961673/GYS2=2}	1.93	1.93	0.23	0.65	1.30	1.19	1.93	6.00	11.00	0.00
{8034783/ADGRL1=1}	{8033825/COL5A3=2}	1.89	1.89	0.23	0.65	1.30	1.19	1.89	6.00	11.00	0.00
{8034940/NOTCH3=1}	{7981142/CLMN=2}	1.79	1.79	0.63	0.85	2.71	1.45	1.79	6.00	11.00	1.00
{8038261/GYS1=1}	{8101992/SLC39A8=1}	1.96	1.96	0.43	0.79	1.75	1.26	1.96	6.00	11.00	0.00
{8042942/HK2=1}	{8146000/ADAM9=1}	1.59	1.59	0.49	0.79	1.94	1.35	1.59	6.00	11.00	0.00
{8042942/HK2=1}	{8094679/KLB=2}	1.74	1.74	0.53	0.79	2.14	1.45	1.74	6.00	11.00	0.00
{8060325/DEFB132=1}	{8048432/CYP27A1=1}	1.98	1.98	0.57	0.79	2.33	1.57	6.00	6.00	11.00	0.00
{8053484/ST3GAL5=2}	{8048432/CYP27A1=1}	1.83	1.83	0.57	0.79	2.33	1.57	1.83	6.00	11.00	0.00

{8053406/RETSAT=1}	{8146000/ADAM9=1}	1.90	1.90	0.49	0.79	1.94	1.35	1.90	6.00	11.00	0.00
{8053406/RETSAT=1}	{8094679/KLB=2}	1.89	1.89	0.53	0.79	2.14	1.45	1.89	6.00	11.00	0.00
{8060325/DEFB132=1}	{7948344/GLYAT=2}	1.95	1.95	0.57	0.79	2.33	1.57	6.00	1.20	11.00	0.00
{8060325/DEFB132=1}	{8094679/KLB=2}	1.97	1.97	0.53	0.79	2.14	1.45	6.00	1.20	11.00	0.00
{8067233/PMEPA1=1}	{8146000/ADAM9=1}	1.50	1.50	0.63	0.85	2.71	1.45	1.50	1.20	11.00	0.00
{8067233/PMEPA1=1}	{8160297/PLIN2=1}	1.83	1.83	0.69	0.85	3.25	1.69	6.00	1.20	11.00	0.00
{8067233/PMEPA1=1}	{7981142/CLMN=2}	1.86	1.86	0.63	0.85	2.71	1.45	1.86	1.20	11.00	0.00
{8071806/DDT|DDTL|LOC391322=1}	{8101992/SLC39A8=1}	1.94	1.94	0.78	0.92	4.50	1.47	1.94	1.20	11.00	0.00
{8074388/SLC25A1=1}	{8101992/SLC39A8=1}	1.84	1.84	0.43	0.79	1.75	1.26	1.84	1.20	11.00	0.00
{8074388/SLC25A1=1}	{8146000/ADAM9=1}	1.83	1.83	0.49	0.79	1.94	1.35	1.83	1.20	11.00	0.00
{8079746/TCTA=1}	{8146000/ADAM9=1}	1.83	1.83	0.25	0.69	1.33	1.18	1.83	1.20	11.00	0.00
{8090852/AMOTL2|RPL39P5=1}	{8141094/PDK4=1}	1.75	1.75	0.36	0.73	1.56	1.26	1.75	1.20	11.00	0.00
{8090852/AMOTL2|RPL39P5=1}	{8180268/CYP51A1=1}	1.82	1.82	0.42	0.73	1.72	1.35	1.82	6.00	11.00	0.00
{8109305/SYNPO=1}	{8101992/SLC39A8=1}	1.90	1.90	0.29	0.73	1.41	1.17	1.90	1.20	11.00	0.00
{8116649/TUBB2A=1}	{8101992/SLC39A8=1}	1.94	1.94	0.43	0.79	1.75	1.26	1.94	6.00	11.00	0.00
{8116653/TUBB2A=1}	{8101992/SLC39A8=1}	1.94	1.94	0.43	0.79	1.75	1.26	1.94	6.00	11.00	0.00
{8131326/SLC29A4=1}	{8101992/SLC39A8=1}	1.88	1.88	0.43	0.79	1.75	1.26	1.88	1.20	11.00	0.00
{8153684/DGAT1=1}	{8101992/SLC39A8=1}	1.85	1.85	0.33	0.75	1.50	1.20	1.85	6.00	12.00	0.00
{8153939/DGAT1=1}	{8101992/SLC39A8=1}	1.85	1.85	0.33	0.75	1.50	1.20	1.85	6.00	12.00	0.00

{7895472/=2}	{8101992/SLC39A8=1}	6.00	6.00	0.29	0.73	1.41	1.17	6.00	1.20	11.00	0.00
{7928516/SAMD8=2}	{8101992/SLC39A8=1}	1.88	1.88	0.59	0.85	2.44	1.35	1.88	1.20	11.00	0.00
{7929201/BTAF1=2}	{8101992/SLC39A8=1}	1.94	1.94	0.43	0.79	1.75	1.26	1.94	1.20	11.00	1.00
{7940153/FAM111A=2}	{8101992/SLC39A8=1}	1.94	1.94	0.62	0.86	2.63	1.37	1.94	1.20	12.00	0.00
{7954419/ETNK1=2}	{8101992/SLC39A8=1}	1.93	1.93	0.78	0.92	4.50	1.47	1.93	6.00	11.00	0.00
{7968761/NAA16=2}	{8101992/SLC39A8=1}	1.94	1.94	0.43	0.79	1.75	1.26	1.94	1.20	11.00	0.00
{7995574/HNRNPA1|HNRNPA1L2|HNRNPA1P10|HNRNPA1P33|XKRX=2}	{8101992/SLC39A8=1}	1.90	1.87	0.59	0.85	2.44	1.35	1.90	1.20	11.00	0.00
{8016745/SPAG9=2}	{8101992/SLC39A8=1}	1.89	1.89	0.59	0.85	2.44	1.35	1.89	1.20	11.00	0.00
{8022814/HNRNPA1|HNRNPA1L2|HNRNPA1P10|HNRNPA1P33|HNRNPA1P6|XKRX=2}	{8101992/SLC39A8=1}	1.90	1.87	0.59	0.85	2.44	1.35	1.90	1.20	11.00	0.00
{8034313/HNRNPA1|HNRNPA1L2|HNRNPA1P10|HNRNPA1P33|HNRNPA1P6|XKRX=2}	{8101992/SLC39A8=1}	1.90	1.87	0.59	0.85	2.44	1.35	1.90	1.20	11.00	0.00
{8038942/ZNF432=2}	{8101992/SLC39A8=1}	1.94	1.94	0.78	0.92	4.50	1.47	1.94	1.20	11.00	0.00
{8051622/SRSF7=2}	{8101992/SLC39A8=1}	1.92	1.92	0.29	0.73	1.41	1.17	1.92	6.00	11.00	0.00
{8055426/MCM6=2}	{8101992/SLC39A8=1}	1.94	1.94	0.47	0.80	1.88	1.28	1.94	6.00	12.00	0.00
{8106141/FCHO2=2}	{8101992/SLC39A8=1}	1.90	1.90	0.47	0.80	1.88	1.28	1.90	1.20	12.00	0.00
{8129045/HDAC2=2}	{8101992/SLC39A8=1}	1.93	1.93	0.79	0.92	4.88	1.48	1.93	6.00	12.00	1.00
{8147019/ZC2HC1A=2}	{8101992/SLC39A8=1}	6.00	6.00	0.59	0.85	2.44	1.35	1.99	1.20	11.00	0.00
{8154531/DENND4C=2}	{8101992/SLC39A8=1}	1.89	1.89	0.59	0.85	2.44	1.35	1.89	1.20	11.00	0.00
{8157216/UGCG=2}	{8101992/SLC39A8=1}	1.89	1.89	0.59	0.85	2.44	1.35	1.89	6.00	11.00	0.00

{8180257/CEP170=2}	{8101992/SLC39A8=1}	6.00	1.94	0.43	0.79	1.75	1.26	1.94	1.20	11.00	0.00
{8115327/SPARC=1}	{8141094/PDK4=1}	1.68	1.68	0.49	0.79	1.94	1.35	1.68	1.20	11.00	0.00
{8119466/MDFI=1}	{8160297/PLIN2=1}	1.83	1.83	0.83	0.92	6.00	1.83	6.00	1.20	11.00	0.00
{8127854/ME1=1}	{7948344/GLYAT=2}	1.80	1.80	0.83	0.92	6.00	1.83	1.80	6.00	11.00	0.00
{8131326/SLC29A4=1}	{8146000/ADAM9=1}	1.89	1.89	0.49	0.79	1.94	1.35	1.89	1.20	11.00	0.00
{8146863/SULF1=2}	{8138466/MACC1=1}	1.54	1.54	0.66	0.85	2.98	1.56	1.54	1.20	11.00	0.00
{8153684/DGAT1=1}	{8141094/PDK4=1}	1.71	1.71	0.40	0.75	1.67	1.29	1.71	6.00	12.00	0.00
{8153939/DGAT1=1}	{8141094/PDK4=1}	1.71	1.71	0.40	0.75	1.67	1.29	1.71	6.00	12.00	0.00
{8180257/CEP170=2}	{8141094/PDK4=1}	6.00	1.88	0.66	0.86	2.92	1.47	1.88	1.20	12.00	0.00
{8153684/DGAT1=1}	{8146000/ADAM9=1}	1.76	1.76	0.25	0.69	1.33	1.18	1.76	6.00	11.00	0.00
{8153939/DGAT1=1}	{8146000/ADAM9=1}	1.76	1.76	0.25	0.69	1.33	1.18	1.76	6.00	11.00	0.00
{8158317/SPTAN1=1}	{8146000/ADAM9=1}	1.52	1.52	0.36	0.73	1.56	1.26	1.52	6.00	11.00	0.00
{8053484/ST3GAL5=2}	{8146000/ADAM9=1}	1.81	1.81	0.66	0.86	2.92	1.47	1.81	6.00	12.00	0.00
{8106141/FCHO2=2}	{8146000/ADAM9=1}	1.84	1.84	0.36	0.73	1.56	1.26	1.84	1.20	11.00	0.00
{8140556/HGF=2}	{8146000/ADAM9=1}	1.00	1.00	0.49	0.79	1.94	1.35	1.00	6.00	11.00	0.00
{8153684/DGAT1=1}	{7961673/GYS2=2}	1.83	1.83	0.32	0.69	1.47	1.27	1.83	6.00	11.00	0.00
{8153684/DGAT1=1}	{7981142/CLMN=2}	1.88	1.88	0.25	0.69	1.33	1.18	1.88	6.00	11.00	0.00
{8153684/DGAT1=1}	{8094679/KLB=2}	1.80	1.80	0.32	0.69	1.47	1.27	1.80	6.00	11.00	0.00
{8153939/DGAT1=1}	{7961673/GYS2=2}	1.83	1.83	0.32	0.69	1.47	1.27	1.83	6.00	11.00	0.00

{8153939/DGAT1=1}	{7981142/CLMN=2}	1.88	1.88	0.25	0.69	1.33	1.18	1.88	6.00	11.00	0.00
{8153939/DGAT1=1}	{8094679/KLB=2}	1.80	1.80	0.32	0.69	1.47	1.27	1.80	6.00	11.00	0.00
{8053484/ST3GAL5=2}	{8160297/PLIN2=1}	1.88	1.88	0.57	0.79	2.33	1.57	6.00	6.00	11.00	0.00
{8101304/RASGEF1B=2}	{8160297/PLIN2=1}	1.91	1.91	1.00	1.00	Inf	2.00	6.00	1.20	11.00	0.00
{8106252/HEXB=2}	{8160297/PLIN2=1}	1.80	1.80	1.00	1.00	Inf	2.00	6.00	6.00	11.00	0.00
{7915207/PABPC4=2}	{8033825/COL5A3=2}	1.86	1.86	0.66	0.85	2.98	1.56	1.86	6.00	11.00	0.00
{8106141/FCHO2=2}	{7919984/SELENBP1=2}	1.86	1.86	0.51	0.73	2.03	1.60	1.86	1.20	11.00	0.00
{7923141/DENND1B=2}	{8033825/COL5A3=2}	1.86	1.86	0.66	0.85	2.98	1.56	1.86	6.00	11.00	0.00
{8140468/GSAP=2}	{7976795/MEG3=2}	6.00	6.00	0.49	0.79	1.94	1.35	6.00	1.20	11.00	0.00
{8053484/ST3GAL5=2}	{7981142/CLMN=2}	1.92	1.92	0.49	0.79	1.94	1.35	1.92	6.00	11.00	0.00
{8102006/MANBA=2}	{7981142/CLMN=2}	1.93	1.93	0.63	0.85	2.71	1.45	1.93	6.00	11.00	0.00
{8180257/CEP170=2}	{7981142/CLMN=2}	6.00	1.94	0.49	0.79	1.94	1.35	1.94	1.20	11.00	0.00
{8102006/MANBA=2}	{8033825/COL5A3=2}	1.84	1.84	0.66	0.85	2.98	1.56	1.84	6.00	11.00	0.00
{8129649/SLC18B1=2}	{8033825/COL5A3=2}	1.97	1.97	0.53	0.79	2.14	1.45	1.97	6.00	11.00	0.00
{8154531/DENND4C=2}	{8033825/COL5A3=2}	1.88	1.88	0.66	0.85	2.98	1.56	1.88	6.00	11.00	0.00
{8180257/CEP170=2}	{8094679/KLB=2}	6.00	1.95	0.53	0.79	2.14	1.45	1.95	1.20	11.00	0.00
{8180257/CEP170=2}	{8121405/C6orf183=2}	6.00	6.00	0.49	0.79	1.94	1.35	6.00	1.20	11.00	0.00
{8140468/GSAP=2}	{8171172/MXRA5=2}	1.97	1.97	0.49	0.79	1.94	1.35	1.97	1.20	11.00	0.00
{7897068/SKI=1,7912692/HSPB7=1}	{8146000/ADAM9=1}	1.90	1.90	0.80	0.92	5.00	1.57	1.90	1.20	11.00	1.00

{7897068/SKI=1,7959298/TMEM120B=1}	{8146000/ADAM9=1}	1.91	1.91	1.00	1.00	Inf	1.71	1.91	1.20	12.00	1.00
{7897068/SKI=1,8023995/FSTL3=1}	{8146000/ADAM9=1}	1.58	1.58	1.00	1.00	Inf	1.71	1.58	1.20	11.00	1.00
{7897068/SKI=1,8034544/PRDX2=1}	{8146000/ADAM9=1}	1.88	1.88	0.80	0.92	5.00	1.57	1.88	1.20	11.00	1.00
{7897068/SKI=1,8053406/RETSAT=1}	{8146000/ADAM9=1}	1.92	1.92	0.80	0.92	5.00	1.57	1.92	6.00	11.00	1.00
{7897068/SKI=1,8067233/PMEPA1=1}	{8146000/ADAM9=1}	1.62	1.62	0.80	0.92	5.00	1.57	1.62	1.20	11.00	1.00
{7897068/SKI=1,8074388/SLC25A1=1}	{8146000/ADAM9=1}	1.92	1.92	1.00	1.00	Inf	1.71	1.92	1.20	11.00	1.00
{7897068/SKI=1,8079746/TCTA=1}	{8146000/ADAM9=1}	1.87	1.87	0.80	0.92	5.00	1.57	1.87	1.20	11.00	1.00
{7897068/SKI=1,8131326/SLC29A4=1}	{8146000/ADAM9=1}	1.96	1.96	1.00	1.00	Inf	1.71	1.96	1.20	11.00	1.00
{7897068/SKI=1,8153684/DGAT1=1}	{8146000/ADAM9=1}	1.87	1.87	0.80	0.92	5.00	1.57	1.87	6.00	11.00	1.00
{7897068/SKI=1,8153939/DGAT1=1}	{8146000/ADAM9=1}	1.87	1.87	0.80	0.92	5.00	1.57	1.87	6.00	11.00	1.00
{7897068/SKI=1,7912692/HSPB7=1}	{7981142/CLMN=2}	1.92	1.92	0.80	0.92	5.00	1.57	1.92	1.20	11.00	1.00
{7897068/SKI=1,7959298/TMEM120B=1}	{7981142/CLMN=2}	1.91	1.91	0.80	0.92	5.00	1.57	1.91	1.20	11.00	1.00
{7897068/SKI=1,8067233/PMEPA1=1}	{7981142/CLMN=2}	1.89	1.89	0.80	0.92	5.00	1.57	1.89	1.20	11.00	1.00
{7899173/DHDDS=1,7948344/GLYAT=1}	{7948344/GLYAT=2}	1.83	1.83	1.00	1.00	Inf	2.00	1.83	6.00	11.00	0.00
{7899173/DHDDS=1,8016832/MMD=1}	{7948344/GLYAT=2}	1.84	1.84	0.69	0.85	3.25	1.69	1.84	6.00	11.00	0.00
{7899173/DHDDS=1,8023995/FSTL3=1}	{7948344/GLYAT=2}	1.85	1.85	0.57	0.79	2.33	1.57	1.85	6.00	11.00	0.00
{7899173/DHDDS=1,8127854/ME1=1}	{7948344/GLYAT=2}	1.85	1.85	0.83	0.92	6.00	1.83	1.85	6.00	11.00	0.00
{7899173/DHDDS=1,8053406/RETSAT=1}	{8094679/KLB=2}	1.90	1.90	0.66	0.85	2.98	1.56	1.90	6.00	11.00	0.00
{7912056/PLEKHG5|TNFRSF25=1,8011188/TLCD2=1}	{8180268/CYP51A1=1}	6.00	1.97	1.00	1.00	Inf	1.85	6.00	6.00	11.00	0.00

{7912692/HSPB7=1,8115327/SPARC=1}	{8141094/PDK4=1}	1.83	1.83	1.00	1.00	Inf	1.71	1.83	1.20	11.00	0.00
{7912692/HSPB7=1,8153684/DGAT1=1}	{8141094/PDK4=1}	1.88	1.88	0.80	0.92	5.00	1.57	1.88	6.00	11.00	0.00
{7912692/HSPB7=1,8153939/DGAT1=1}	{8141094/PDK4=1}	1.88	1.88	0.80	0.92	5.00	1.57	1.88	6.00	11.00	0.00
{7959298/TMEM120B=1,7912692/HSPB7=1}	{8146000/ADAM9=1}	1.94	1.94	1.00	1.00	Inf	1.71	1.94	1.20	11.00	0.00
{7912692/HSPB7=1,8067233/PMEPA1=1}	{8146000/ADAM9=1}	1.91	1.91	0.80	0.92	5.00	1.57	1.91	1.20	11.00	0.00
{7959298/TMEM120B=1,7912692/HSPB7=1}	{7981142/CLMN=2}	1.94	1.94	1.00	1.00	Inf	1.71	1.94	1.20	11.00	0.00
{7912692/HSPB7=1,8067233/PMEPA1=1}	{7981142/CLMN=2}	1.93	1.93	0.80	0.92	5.00	1.57	1.93	1.20	11.00	0.00
{7928872/SNCG=1,8109305/SYNPO=1}	{8101992/SLC39A8=1}	1.93	1.93	0.43	0.79	1.75	1.26	1.93	1.20	11.00	0.00
{7936322/GPAM=1,8119466/MDFI=1}	{8160297/PLIN2=1}	1.84	1.84	0.83	0.92	6.00	1.83	6.00	6.00	11.00	0.00
{8016832/MMD=1,7948344/GLYAT=1}	{7948344/GLYAT=2}	1.84	1.84	1.00	1.00	Inf	2.00	1.84	1.20	11.00	0.00
{8023995/FSTL3=1,7948344/GLYAT=1}	{7948344/GLYAT=2}	1.78	1.78	1.00	1.00	Inf	2.00	1.78	1.20	11.00	0.00
{8127854/ME1=1,7948344/GLYAT=1}	{7948344/GLYAT=2}	1.80	1.80	1.00	1.00	Inf	2.00	1.80	6.00	11.00	0.00
{8023995/FSTL3=1,7959298/TMEM120B=1}	{8146000/ADAM9=1}	1.92	1.92	0.80	0.92	5.00	1.57	1.92	1.20	11.00	0.00
{7959298/TMEM120B=1,8034544/PRDX2=1}	{8146000/ADAM9=1}	1.94	1.94	1.00	1.00	Inf	1.71	1.94	1.20	11.00	0.00
{7959298/TMEM120B=1,8053406/RETSAT=1}	{8146000/ADAM9=1}	1.94	1.94	1.00	1.00	Inf	1.71	1.94	6.00	11.00	0.00
{7959298/TMEM120B=1,8067233/PMEPA1=1}	{8146000/ADAM9=1}	1.90	1.90	0.80	0.92	5.00	1.57	1.90	1.20	11.00	0.00
{7959298/TMEM120B=1,8074388/SLC25A1=1}	{8146000/ADAM9=1}	1.93	1.93	1.00	1.00	Inf	1.71	1.93	1.20	11.00	0.00
{7959298/TMEM120B=1,8079746/TCTA=1}	{8146000/ADAM9=1}	1.92	1.92	0.80	0.92	5.00	1.57	1.92	1.20	11.00	0.00
{7959298/TMEM120B=1,8131326/SLC29A4=1}	{8146000/ADAM9=1}	1.94	1.94	1.00	1.00	Inf	1.71	1.94	1.20	11.00	0.00

{7959298/TMEM120B=1,8153684/DGAT1=1}	{8146000/ADAM9=1}	1.90	1.90	1.00	1.00	Inf	1.71	1.90	6.00	11.00	0.00
{7959298/TMEM120B=1,8153939/DGAT1=1}	{8146000/ADAM9=1}	1.90	1.90	1.00	1.00	Inf	1.71	1.90	6.00	11.00	0.00
{7959298/TMEM120B=1,8067233/PMEPA1=1}	{8160297/PLIN2=1}	1.93	1.93	0.83	0.92	6.00	1.83	6.00	1.20	11.00	0.00
{7959298/TMEM120B=1,8067233/PMEPA1=1}	{7981142/CLMN=2}	1.90	1.90	0.80	0.92	5.00	1.57	1.90	1.20	11.00	0.00
{8016033/FAM171A2=1,8034544/PRDX2=1}	{8101992/SLC39A8=1}	6.00	1.99	0.29	0.73	1.41	1.17	6.00	1.20	11.00	0.00
{8023995/FSTL3=1,8016832/MMD=1}	{7948344/GLYAT=2}	1.86	1.86	0.69	0.85	3.25	1.69	1.86	1.20	11.00	0.00
{8127854/ME1=1,8016832/MMD=1}	{7948344/GLYAT=2}	1.86	1.86	1.00	1.00	Inf	2.00	1.86	6.00	11.00	0.00
{8023995/FSTL3=1,8053406/RETSAT=1}	{8146000/ADAM9=1}	1.92	1.92	0.63	0.85	2.71	1.45	1.92	6.00	11.00	0.00
{8023995/FSTL3=1,8131326/SLC29A4=1}	{8146000/ADAM9=1}	1.97	1.97	0.63	0.85	2.71	1.45	1.97	1.20	11.00	0.00
{8023995/FSTL3=1,8127854/ME1=1}	{7948344/GLYAT=2}	1.86	1.86	1.00	1.00	Inf	2.00	1.86	6.00	11.00	0.00
{8024323/REEP6=1,8034544/PRDX2=1}	{8101992/SLC39A8=1}	1.95	1.95	0.59	0.85	2.44	1.35	1.95	1.20	11.00	0.00
{8024323/REEP6=1,8074388/SLC25A1=1}	{8101992/SLC39A8=1}	1.91	1.91	0.59	0.85	2.44	1.35	1.91	1.20	11.00	0.00
{8024323/REEP6=1,8153684/DGAT1=1}	{8101992/SLC39A8=1}	1.90	1.90	0.47	0.80	1.88	1.28	1.90	6.00	12.00	0.00
{8024323/REEP6=1,8153939/DGAT1=1}	{8101992/SLC39A8=1}	1.90	1.90	0.47	0.80	1.88	1.28	1.90	6.00	12.00	0.00
{8024323/REEP6=1,8153684/DGAT1=1}	{8141094/PDK4=1}	1.87	1.87	0.36	0.73	1.56	1.26	1.87	6.00	11.00	0.00
{8024323/REEP6=1,8153939/DGAT1=1}	{8141094/PDK4=1}	1.87	1.87	0.36	0.73	1.56	1.26	1.87	6.00	11.00	0.00
{8024323/REEP6=1,8180257/CEP170=2}	{8141094/PDK4=1}	6.00	1.90	0.63	0.85	2.71	1.45	1.90	1.20	11.00	0.00
{8024323/REEP6=1,8153684/DGAT1=1}	{8094679/KLB=2}	1.91	1.91	0.42	0.73	1.72	1.35	1.91	6.00	11.00	0.00
{8024323/REEP6=1,8153939/DGAT1=1}	{8094679/KLB=2}	1.91	1.91	0.42	0.73	1.72	1.35	1.91	6.00	11.00	0.00

{8034544/PRDX2=1,8074388/SLC25A1=1}	{8101992/SLC39A8=1}	1.95	1.95	0.59	0.85	2.44	1.35	1.95	1.20	11.00	0.00
{8131326/SLC29A4=1,8034544/PRDX2=1}	{8101992/SLC39A8=1}	1.97	1.97	0.59	0.85	2.44	1.35	1.97	1.20	11.00	0.00
{8153684/DGAT1=1,8034544/PRDX2=1}	{8101992/SLC39A8=1}	1.93	1.93	0.43	0.79	1.75	1.26	1.93	6.00	11.00	0.00
{8034544/PRDX2=1,8153939/DGAT1=1}	{8101992/SLC39A8=1}	1.93	1.93	0.43	0.79	1.75	1.26	1.93	6.00	11.00	0.00
{8153684/DGAT1=1,8034783/ADGRL1=1}	{8141094/PDK4=1}	1.91	1.91	0.49	0.79	1.94	1.35	1.91	6.00	11.00	0.00
{8034783/ADGRL1=1,8153939/DGAT1=1}	{8141094/PDK4=1}	1.91	1.91	0.49	0.79	1.94	1.35	1.91	6.00	11.00	0.00
{8131326/SLC29A4=1,8053406/RETSAT=1}	{8146000/ADAM9=1}	1.96	1.96	0.63	0.85	2.71	1.45	1.96	6.00	11.00	0.00
{8153684/DGAT1=1,8074388/SLC25A1=1}	{8101992/SLC39A8=1}	1.89	1.89	0.43	0.79	1.75	1.26	1.89	6.00	11.00	0.00
{8153939/DGAT1=1,8074388/SLC25A1=1}	{8101992/SLC39A8=1}	1.89	1.89	0.43	0.79	1.75	1.26	1.89	6.00	11.00	0.00
{8153684/DGAT1=1,8074388/SLC25A1=1}	{8146000/ADAM9=1}	1.87	1.87	0.49	0.79	1.94	1.35	1.87	6.00	11.00	0.00
{8153939/DGAT1=1,8074388/SLC25A1=1}	{8146000/ADAM9=1}	1.87	1.87	0.49	0.79	1.94	1.35	1.87	6.00	11.00	0.00
{8116653/TUBB2A=1,8116649/TUBB2A=1}	{8101992/SLC39A8=1}	1.94	1.94	0.43	0.79	1.75	1.26	1.94	6.00	11.00	0.00
{8153684/DGAT1=1,8153939/DGAT1=1}	{8101992/SLC39A8=1}	1.85	1.85	0.33	0.75	1.50	1.20	1.85	6.00	12.00	0.00
{8022814/HNRNPA1|HNRNPA1L2|HNRNPA1P10|HNRNPA1P33|HNRNPA1P6|XKRX=2,7928516/SAMD8=2}	{8101992/SLC39A8=1}	1.93	1.90	0.78	0.92	4.50	1.47	1.93	1.20	11.00	0.00
{7929201/BTAF1=2,7940153/FAM111A=2}	{8101992/SLC39A8=1}	1.94	1.94	0.78	0.92	4.50	1.47	1.94	1.20	11.00	1.00
{7929201/BTAF1=2,8106141/FCHO2=2}	{8101992/SLC39A8=1}	1.95	1.95	0.59	0.85	2.44	1.35	1.95	1.20	11.00	1.00
{7954419/ETNK1=2,7940153/FAM111A=2}	{8101992/SLC39A8=1}	1.94	1.94	1.00	1.00	Inf	1.60	1.94	6.00	11.00	0.00
{7940153/FAM111A=2,8106141/FCHO2=2}	{8101992/SLC39A8=1}	1.95	1.95	0.79	0.92	4.88	1.48	1.95	1.20	12.00	0.00

{7954419/ETNK1=2,8106141/FCHO2=2}	{8101992/SLC39A8=1}	1.93	1.93	0.78	0.92	4.50	1.47	1.93	6.00	11.00	0.00
{8034313/HNRNPA1|HNRNPA1L2|HNRNPA1P10|HNRNPA1P33|HNRNPA1P6|XKRX=2,7995574/HNRNPA1|HNRNPA1L2|HNRNPA1P10|HNRNPA1P33|XKRX=2}	{8101992/SLC39A8=1}	1.90	1.87	0.59	0.85	2.44	1.35	1.90	1.20	11.00	0.00
{8153684/DGAT1=1,8115327/SPARC=1}	{8141094/PDK4=1}	1.86	1.86	0.80	0.92	5.00	1.57	1.86	6.00	11.00	0.00
{8115327/SPARC=1,8153939/DGAT1=1}	{8141094/PDK4=1}	1.86	1.86	0.80	0.92	5.00	1.57	1.86	6.00	11.00	0.00
{8153684/DGAT1=1,8153939/DGAT1=1}	{8141094/PDK4=1}	1.71	1.71	0.40	0.75	1.67	1.29	1.71	6.00	12.00	0.00
{8153684/DGAT1=1,8180257/CEP170=2}	{8141094/PDK4=1}	6.00	1.89	0.63	0.85	2.71	1.45	1.89	6.00	11.00	0.00
{8153939/DGAT1=1,8180257/CEP170=2}	{8141094/PDK4=1}	6.00	1.89	0.63	0.85	2.71	1.45	1.89	6.00	11.00	0.00
{8153684/DGAT1=1,8153939/DGAT1=1}	{8146000/ADAM9=1}	1.76	1.76	0.25	0.69	1.33	1.18	1.76	6.00	11.00	0.00
{8153684/DGAT1=1,8153939/DGAT1=1}	{7961673/GYS2=2}	1.83	1.83	0.32	0.69	1.47	1.27	1.83	6.00	11.00	0.00
{8153684/DGAT1=1,8153939/DGAT1=1}	{7981142/CLMN=2}	1.88	1.88	0.25	0.69	1.33	1.18	1.88	6.00	11.00	0.00
{8153684/DGAT1=1,8153939/DGAT1=1}	{8094679/KLB=2}	1.80	1.80	0.32	0.69	1.47	1.27	1.80	6.00	11.00	0.00
{8129649/SLC18B1=2,8154531/DENND4C=2}	{8033825/COL5A3=2}	1.97	1.97	0.82	0.92	5.50	1.69	1.97	6.00	11.00	0.00
{7897068/SKI=1,7959298/TMEM120B=1,7912692/HSPB7=1}	{8146000/ADAM9=1}	1.94	1.94	1.00	1.00	Inf	1.71	1.94	1.20	11.00	1.00
{7897068/SKI=1,7912692/HSPB7=1,8067233/PMEPA1=1}	{8146000/ADAM9=1}	1.91	1.91	0.80	0.92	5.00	1.57	1.91	1.20	11.00	1.00
{7897068/SKI=1,8023995/FSTL3=1,7959298/TMEM120B=1}	{8146000/ADAM9=1}	1.92	1.92	1.00	1.00	Inf	1.71	1.92	1.20	11.00	1.00
{7897068/SKI=1,7959298/TMEM120B=1,8034544/PRDX2=1}	{8146000/ADAM9=1}	1.94	1.94	1.00	1.00	Inf	1.71	1.94	1.20	11.00	1.00
{7897068/SKI=1,7959298/TMEM120B=1,8053406/RETSAT=1}	{8146000/ADAM9=1}	1.95	1.95	1.00	1.00	Inf	1.71	1.95	6.00	11.00	1.00

{7897068/SKI=1,7959298/TMEM120B=1,8067233/PMEPA1=1}	{8146000/ADAM9=1}	1.93	1.93	1.00	1.00	Inf	1.71	1.93	1.20	11.00	1.00
{7897068/SKI=1,7959298/TMEM120B=1,8074388/SLC25A1=1}	{8146000/ADAM9=1}	1.95	1.95	1.00	1.00	Inf	1.71	1.95	1.20	11.00	1.00
{7897068/SKI=1,7959298/TMEM120B=1,8079746/TCTA=1}	{8146000/ADAM9=1}	1.94	1.94	1.00	1.00	Inf	1.71	1.94	1.20	11.00	1.00
{7897068/SKI=1,7959298/TMEM120B=1,8131326/SLC29A4=1}	{8146000/ADAM9=1}	1.97	1.97	1.00	1.00	Inf	1.71	1.97	1.20	11.00	1.00
{7897068/SKI=1,7959298/TMEM120B=1,8153684/DGAT1=1}	{8146000/ADAM9=1}	1.93	1.93	1.00	1.00	Inf	1.71	1.93	6.00	11.00	1.00
{7897068/SKI=1,7959298/TMEM120B=1,8153939/DGAT1=1}	{8146000/ADAM9=1}	1.93	1.93	1.00	1.00	Inf	1.71	1.93	6.00	11.00	1.00
{7897068/SKI=1,8023995/FSTL3=1,8053406/RETSAT=1}	{8146000/ADAM9=1}	1.92	1.92	1.00	1.00	Inf	1.71	1.92	6.00	11.00	1.00
{7897068/SKI=1,8023995/FSTL3=1,8131326/SLC29A4=1}	{8146000/ADAM9=1}	1.97	1.97	1.00	1.00	Inf	1.71	1.97	1.20	11.00	1.00
{7897068/SKI=1,8131326/SLC29A4=1,8053406/RETSAT=1}	{8146000/ADAM9=1}	1.97	1.97	1.00	1.00	Inf	1.71	1.97	6.00	11.00	1.00
{7897068/SKI=1,8153684/DGAT1=1,8074388/SLC25A1=1}	{8146000/ADAM9=1}	1.92	1.92	1.00	1.00	Inf	1.71	1.92	6.00	11.00	1.00
{7897068/SKI=1,8153939/DGAT1=1,8074388/SLC25A1=1}	{8146000/ADAM9=1}	1.92	1.92	1.00	1.00	Inf	1.71	1.92	6.00	11.00	1.00
{7897068/SKI=1,8153684/DGAT1=1,8153939/DGAT1=1}	{8146000/ADAM9=1}	1.87	1.87	0.80	0.92	5.00	1.57	1.87	6.00	11.00	1.00
{7897068/SKI=1,7959298/TMEM120B=1,7912692/HSPB7=1}	{7981142/CLMN=2}	1.94	1.94	1.00	1.00	Inf	1.71	1.94	1.20	11.00	1.00
{7897068/SKI=1,7912692/HSPB7=1,8067233/PMEPA1=1}	{7981142/CLMN=2}	1.93	1.93	0.80	0.92	5.00	1.57	1.93	1.20	11.00	1.00
{7897068/SKI=1,7959298/TMEM120B=1,8067233/PMEPA1=1}	{7981142/CLMN=2}	1.93	1.93	1.00	1.00	Inf	1.71	1.93	1.20	11.00	1.00
{7899173/DHDDS=1,8016832/MMD=1,7948344/GLYAT=1}	{7948344/GLYAT=2}	1.84	1.84	1.00	1.00	Inf	2.00	1.84	6.00	11.00	0.00

{7899173/DHDDS=1,8023995/FSTL3=1,7948344/GLYAT=1}	{7948344/GLYAT=2}	1.85	1.85	1.00	1.00	Inf	2.00	1.85	6.00	11.00	0.00
{7899173/DHDDS=1,8127854/ME1=1,7948344/GLYAT=1}	{7948344/GLYAT=2}	1.85	1.85	1.00	1.00	Inf	2.00	1.85	6.00	11.00	0.00
{7899173/DHDDS=1,8023995/FSTL3=1,8016832/MMD=1}	{7948344/GLYAT=2}	1.86	1.86	0.69	0.85	3.25	1.69	1.86	6.00	11.00	0.00
{7899173/DHDDS=1,8127854/ME1=1,8016832/MMD=1}	{7948344/GLYAT=2}	1.86	1.86	1.00	1.00	Inf	2.00	1.86	6.00	11.00	0.00
{7899173/DHDDS=1,8023995/FSTL3=1,8127854/ME1=1}	{7948344/GLYAT=2}	1.87	1.87	1.00	1.00	Inf	2.00	1.87	6.00	11.00	0.00
{7912692/HSPB7=1,8153684/DGAT1=1,8115327/SPARC=1}	{8141094/PDK4=1}	1.88	1.88	1.00	1.00	Inf	1.71	1.88	6.00	11.00	0.00
{7912692/HSPB7=1,8115327/SPARC=1,8153939/DGAT1=1}	{8141094/PDK4=1}	1.88	1.88	1.00	1.00	Inf	1.71	1.88	6.00	11.00	0.00
{7912692/HSPB7=1,8153684/DGAT1=1,8153939/DGAT1=1}	{8141094/PDK4=1}	1.88	1.88	0.80	0.92	5.00	1.57	1.88	6.00	11.00	0.00
{7959298/TMEM120B=1,7912692/HSPB7=1,8067233/PMEPA1=1}	{8146000/ADAM9=1}	1.94	1.94	1.00	1.00	Inf	1.71	1.94	1.20	11.00	0.00
{7959298/TMEM120B=1,7912692/HSPB7=1,8067233/PMEPA1=1}	{7981142/CLMN=2}	1.94	1.94	1.00	1.00	Inf	1.71	1.94	1.20	11.00	0.00
{8023995/FSTL3=1,8016832/MMD=1,7948344/GLYAT=1}	{7948344/GLYAT=2}	1.86	1.86	1.00	1.00	Inf	2.00	1.86	1.20	11.00	0.00
{8127854/ME1=1,8016832/MMD=1,7948344/GLYAT=1}	{7948344/GLYAT=2}	1.86	1.86	1.00	1.00	Inf	2.00	1.86	6.00	11.00	0.00
{8023995/FSTL3=1,8127854/ME1=1,7948344/GLYAT=1}	{7948344/GLYAT=2}	1.86	1.86	1.00	1.00	Inf	2.00	1.86	6.00	11.00	0.00
{8023995/FSTL3=1,7959298/TMEM120B=1,8053406/RETSAT=1}	{8146000/ADAM9=1}	1.95	1.95	1.00	1.00	Inf	1.71	1.95	6.00	11.00	0.00
{8023995/FSTL3=1,7959298/TMEM120B=1,8131326/SLC29A4=1}	{8146000/ADAM9=1}	1.97	1.97	1.00	1.00	Inf	1.71	1.97	1.20	11.00	0.00
{7959298/TMEM120B=1,8131326/SLC29A4=1,8053406/RETSAT=1}	{8146000/ADAM9=1}	1.96	1.96	1.00	1.00	Inf	1.71	1.96	6.00	11.00	0.00

{7959298/TMEM120B=1,8153684/DGAT1=1,8074388/SLC25A1=1}	{8146000/ADAM9=1}	1.93	1.93	1.00	1.00	Inf	1.71	1.93	6.00	11.00	0.00
{7959298/TMEM120B=1,8153939/DGAT1=1,8074388/SLC25A1=1}	{8146000/ADAM9=1}	1.93	1.93	1.00	1.00	Inf	1.71	1.93	6.00	11.00	0.00
{7959298/TMEM120B=1,8153684/DGAT1=1,8153939/DGAT1=1}	{8146000/ADAM9=1}	1.90	1.90	1.00	1.00	Inf	1.71	1.90	6.00	11.00	0.00
{8023995/FSTL3=1,8127854/ME1=1,8016832/MMD=1}	{7948344/GLYAT=2}	1.88	1.88	1.00	1.00	Inf	2.00	1.88	6.00	11.00	0.00
{8023995/FSTL3=1,8131326/SLC29A4=1,8053406/RETSAT=1}	{8146000/ADAM9=1}	1.97	1.97	0.63	0.85	2.71	1.45	1.97	6.00	11.00	0.00
{8024323/REEP6=1,8034544/PRDX2=1,8074388/SLC25A1=1}	{8101992/SLC39A8=1}	1.96	1.96	0.78	0.92	4.50	1.47	1.96	1.20	11.00	0.00
{8024323/REEP6=1,8153684/DGAT1=1,8034544/PRDX2=1}	{8101992/SLC39A8=1}	1.95	1.95	0.59	0.85	2.44	1.35	1.95	6.00	11.00	0.00
{8024323/REEP6=1,8034544/PRDX2=1,8153939/DGAT1=1}	{8101992/SLC39A8=1}	1.95	1.95	0.59	0.85	2.44	1.35	1.95	6.00	11.00	0.00
{8024323/REEP6=1,8153684/DGAT1=1,8074388/SLC25A1=1}	{8101992/SLC39A8=1}	1.91	1.91	0.59	0.85	2.44	1.35	1.91	6.00	11.00	0.00
{8024323/REEP6=1,8153939/DGAT1=1,8074388/SLC25A1=1}	{8101992/SLC39A8=1}	1.91	1.91	0.59	0.85	2.44	1.35	1.91	6.00	11.00	0.00
{8024323/REEP6=1,8153684/DGAT1=1,8153939/DGAT1=1}	{8101992/SLC39A8=1}	1.90	1.90	0.47	0.80	1.88	1.28	1.90	6.00	12.00	0.00
{8024323/REEP6=1,8153684/DGAT1=1,8153939/DGAT1=1}	{8141094/PDK4=1}	1.87	1.87	0.36	0.73	1.56	1.26	1.87	6.00	11.00	0.00
{8024323/REEP6=1,8153684/DGAT1=1,8180257/CEP170=2}	{8141094/PDK4=1}	6.00	1.90	0.63	0.85	2.71	1.45	1.90	6.00	11.00	0.00
{8024323/REEP6=1,8153939/DGAT1=1,8180257/CEP170=2}	{8141094/PDK4=1}	6.00	1.90	0.63	0.85	2.71	1.45	1.90	6.00	11.00	0.00
{8024323/REEP6=1,8153684/DGAT1=1,8153939/DGAT1=1}	{8094679/KLB=2}	1.91	1.91	0.42	0.73	1.72	1.35	1.91	6.00	11.00	0.00
{8153684/DGAT1=1,8034544/PRDX2=1,8074388/SLC25A1=1}	{8101992/SLC39A8=1}	1.95	1.95	0.59	0.85	2.44	1.35	1.95	6.00	11.00	0.00

{8034544/PRDX2=1,8153939/DGAT1=1,8074388/SLC25A1=1}	{8101992/SLC39A8=1}	1.95	1.95	0.59	0.85	2.44	1.35	1.95	6.00	11.00	0.00
{8153684/DGAT1=1,8034544/PRDX2=1,8153939/DGAT1=1}	{8101992/SLC39A8=1}	1.93	1.93	0.43	0.79	1.75	1.26	1.93	6.00	11.00	0.00
{8153684/DGAT1=1,8034783/ADGRL1=1,8153939/DGAT1=1}	{8141094/PDK4=1}	1.91	1.91	0.49	0.79	1.94	1.35	1.91	6.00	11.00	0.00
{8153684/DGAT1=1,8153939/DGAT1=1,8074388/SLC25A1=1}	{8101992/SLC39A8=1}	1.89	1.89	0.43	0.79	1.75	1.26	1.89	6.00	11.00	0.00
{8153684/DGAT1=1,8153939/DGAT1=1,8074388/SLC25A1=1}	{8146000/ADAM9=1}	1.87	1.87	0.49	0.79	1.94	1.35	1.87	6.00	11.00	0.00
{7929201/BTAF1=2,7940153/FAM111A=2,8106141/FCHO2=2}	{8101992/SLC39A8=1}	1.95	1.95	0.78	0.92	4.50	1.47	1.95	1.20	11.00	1.00
{7954419/ETNK1=2,7940153/FAM111A=2,8106141/FCHO2=2}	{8101992/SLC39A8=1}	1.95	1.95	1.00	1.00	Inf	1.60	1.95	6.00	11.00	0.00
{8153684/DGAT1=1,8115327/SPARC=1,8153939/DGAT1=1}	{8141094/PDK4=1}	1.86	1.86	0.80	0.92	5.00	1.57	1.86	6.00	11.00	0.00
{8153684/DGAT1=1,8153939/DGAT1=1,8180257/CEP170=2}	{8141094/PDK4=1}	6.00	1.89	0.63	0.85	2.71	1.45	1.89	6.00	11.00	0.00
{7897068/SKI=1,7959298/TMEM120B=1,7912692/HSPB7=1,8067233/PMEPA1=1}	{8146000/ADAM9=1}	1.94	1.94	1.00	1.00	Inf	1.71	1.94	1.20	11.00	1.00
{7897068/SKI=1,8023995/FSTL3=1,7959298/TMEM120B=1,8053406/RETSAT=1}	{8146000/ADAM9=1}	1.95	1.95	1.00	1.00	Inf	1.71	1.95	6.00	11.00	1.00
{7897068/SKI=1,8023995/FSTL3=1,7959298/TMEM120B=1,8131326/SLC29A4=1}	{8146000/ADAM9=1}	1.97	1.97	1.00	1.00	Inf	1.71	1.97	1.20	11.00	1.00
{7897068/SKI=1,7959298/TMEM120B=1,8131326/SLC29A4=1,8053406/RETSAT=1}	{8146000/ADAM9=1}	1.97	1.97	1.00	1.00	Inf	1.71	1.97	6.00	11.00	1.00
{7897068/SKI=1,7959298/TMEM120B=1,8153684/DGAT1=1,8074388/SLC25A1=1}	{8146000/ADAM9=1}	1.95	1.95	1.00	1.00	Inf	1.71	1.95	6.00	11.00	1.00
{7897068/SKI=1,7959298/TMEM120B=1,8153939/DGAT1=1,8074388/SLC25A1=1}	{8146000/ADAM9=1}	1.95	1.95	1.00	1.00	Inf	1.71	1.95	6.00	11.00	1.00
{7897068/SKI=1,7959298/TMEM120B=1,8153684/DGAT1=1,8153939/DGAT1=1}	{8146000/ADAM9=1}	1.93	1.93	1.00	1.00	Inf	1.71	1.93	6.00	11.00	1.00

{7897068/SKI=1,8023995/FSTL3=1,8131326/SLC29A4=1,8053406/RETSAT=1}	{8146000/ADAM9=1}	1.97	1.97	1.00	1.00	Inf	1.71	1.97	6.00	11.00	1.00
{7897068/SKI=1,8153684/DGAT1=1,8153939/DGAT1=1,8074388/SLC25A1=1}	{8146000/ADAM9=1}	1.92	1.92	1.00	1.00	Inf	1.71	1.92	6.00	11.00	1.00
{7897068/SKI=1,7959298/TMEM120B=1,7912692/HSPB7=1,8067233/PMEPA1=1}	{7981142/CLMN=2}	1.94	1.94	1.00	1.00	Inf	1.71	1.94	1.20	11.00	1.00
{7899173/DHDDS=1,8023995/FSTL3=1,8016832/MMD=1,7948344/GLYAT=1}	{7948344/GLYAT=2}	1.86	1.86	1.00	1.00	Inf	2.00	1.86	6.00	11.00	0.00
{7899173/DHDDS=1,8127854/ME1=1,8016832/MMD=1,7948344/GLYAT=1}	{7948344/GLYAT=2}	1.86	1.86	1.00	1.00	Inf	2.00	1.86	6.00	11.00	0.00
{7899173/DHDDS=1,8023995/FSTL3=1,8127854/ME1=1,7948344/GLYAT=1}	{7948344/GLYAT=2}	1.87	1.87	1.00	1.00	Inf	2.00	1.87	6.00	11.00	0.00
{7899173/DHDDS=1,8023995/FSTL3=1,8127854/ME1=1,8016832/MMD=1}	{7948344/GLYAT=2}	1.88	1.88	1.00	1.00	Inf	2.00	1.88	6.00	11.00	0.00
{7912692/HSPB7=1,8153684/DGAT1=1,8115327/SPARC=1,8153939/DGAT1=1}	{8141094/PDK4=1}	1.88	1.88	1.00	1.00	Inf	1.71	1.88	6.00	11.00	0.00
{8023995/FSTL3=1,8127854/ME1=1,8016832/MMD=1,7948344/GLYAT=1}	{7948344/GLYAT=2}	1.88	1.88	1.00	1.00	Inf	2.00	1.88	6.00	11.00	0.00
{8023995/FSTL3=1,7959298/TMEM120B=1,8131326/SLC29A4=1,8053406/RETSAT=1}	{8146000/ADAM9=1}	1.97	1.97	1.00	1.00	Inf	1.71	1.97	6.00	11.00	0.00
{7959298/TMEM120B=1,8153684/DGAT1=1,8153939/DGAT1=1,8074388/SLC25A1=1}	{8146000/ADAM9=1}	1.93	1.93	1.00	1.00	Inf	1.71	1.93	6.00	11.00	0.00
{8024323/REEP6=1,8153684/DGAT1=1,8034544/PRDX2=1,8074388/SLC25A1=1}	{8101992/SLC39A8=1}	1.96	1.96	0.78	0.92	4.50	1.47	1.96	6.00	11.00	0.00
{8024323/REEP6=1,8034544/PRDX2=1,8153939/DGAT1=1,8074388/SLC25A1=1}	{8101992/SLC39A8=1}	1.96	1.96	0.78	0.92	4.50	1.47	1.96	6.00	11.00	0.00
{8024323/REEP6=1,8153684/DGAT1=1,8034544/PRDX2=1,8153939/DGAT1=1}	{8101992/SLC39A8=1}	1.95	1.95	0.59	0.85	2.44	1.35	1.95	6.00	11.00	0.00
{8024323/REEP6=1,8153684/DGAT1=1,8153939/DGAT1=1,8074388/SLC25A1=1}	{8101992/SLC39A8=1}	1.91	1.91	0.59	0.85	2.44	1.35	1.91	6.00	11.00	0.00
{8024323/REEP6=1,8153684/DGAT1=1,8153939/DGAT1=1,8180257/CEP170=2}	{8141094/PDK4=1}	6.00	1.90	0.63	0.85	2.71	1.45	1.90	6.00	11.00	0.00

{8153684/DGAT1=1,8034544/PRDX2=1,8153939/DGAT1=1,8074388/SLC25A1=1}	{8101992/SLC39A8=1}	1.95	1.95	0.59	0.85	2.44	1.35	1.95	6.00	11.00	0.00
{7897068/SKI=1,8023995/FSTL3=1,7959298/TMEM120B=1,8131326/SLC29A4=1,8053406/RETSAT=1}	{8146000/ADAM9=1}	1.97	1.97	1.00	1.00	Inf	1.71	1.97	6.00	11.00	1.00
{7897068/SKI=1,7959298/TMEM120B=1,8153684/DGAT1=1,8153939/DGAT1=1,8074388/SLC25A1=1}	{8146000/ADAM9=1}	1.95	1.95	1.00	1.00	Inf	1.71	1.95	6.00	11.00	1.00
{7899173/DHDDS=1,8023995/FSTL3=1,8127854/ME1=1,8016832/MMD=1,7948344/GLYAT=1}	{7948344/GLYAT=2}	1.88	1.88	1.00	1.00	Inf	2.00	1.88	6.00	11.00	0.00
{8024323/REEP6=1,8153684/DGAT1=1,8034544/PRDX2=1,8153939/DGAT1=1,8074388/SLC25A1=1}	{8101992/SLC39A8=1}	1.96	1.96	0.78	0.92	4.50	1.47	1.96	6.00	11.00	0.00
{7897068/SKI=1}	{7981142/CLMN=2,8146000/ADAM9=1}	1.88	1.88	0.69	0.85	3.25	1.69	1.88	1.20	11.00	1.00
{8042942/HK2=1}	{7907160/ATP1B1=1,8146000/ADAM9=1}	1.74	1.74	0.60	0.79	2.53	1.71	1.74	6.00	11.00	0.00
{7912692/HSPB7=1}	{7981142/CLMN=2,8146000/ADAM9=1}	1.94	1.94	0.69	0.85	3.25	1.69	1.94	1.20	11.00	0.00
{7959298/TMEM120B=1}	{7981142/CLMN=2,8146000/ADAM9=1}	1.89	1.89	0.69	0.85	3.25	1.69	1.89	1.20	11.00	0.00
{8067233/PMEPA1=1}	{7981142/CLMN=2,8146000/ADAM9=1}	1.89	1.89	0.69	0.85	3.25	1.69	1.89	1.20	11.00	0.00
{8180257/CEP170=2}	{8141094/PDK4=1,8094679/KLB=2}	6.00	1.96	0.60	0.79	2.53	1.71	1.96	1.20	11.00	0.00
{8053484/ST3GAL5=2}	{7981142/CLMN=2,8146000/ADAM9=1}	1.94	1.94	0.57	0.79	2.33	1.57	1.94	6.00	11.00	0.00
{7897068/SKI=1,7912692/HSPB7=1}	{7981142/CLMN=2,8146000/ADAM9=1}	1.94	1.94	0.83	0.92	6.00	1.83	1.94	1.20	11.00	1.00
{7897068/SKI=1,7959298/TMEM120B=1}	{7981142/CLMN=2,8146000/ADAM9=1}	1.92	1.92	0.83	0.92	6.00	1.83	1.92	1.20	11.00	1.00

{7897068/SKI=1,8067233/PMEPA1=1}	{7981142/CLMN=2,8146000/ADAM9=1}	1.92	1.92	0.83	0.92	6.00	1.83	1.92	1.20	11.00	1.00
{7959298/TMEM120B=1,7912692/HSPB7=1}	{7981142/CLMN=2,8146000/ADAM9=1}	1.96	1.96	1.00	1.00	Inf	2.00	1.96	1.20	11.00	0.00
{7912692/HSPB7=1,8067233/PMEPA1=1}	{7981142/CLMN=2,8146000/ADAM9=1}	1.94	1.94	0.83	0.92	6.00	1.83	1.94	1.20	11.00	0.00
{7959298/TMEM120B=1,8067233/PMEPA1=1}	{7981142/CLMN=2,8146000/ADAM9=1}	1.91	1.91	0.83	0.92	6.00	1.83	1.91	1.20	11.00	0.00
{7897068/SKI=1,7959298/TMEM120B=1,7912692/HSPB7=1}	{7981142/CLMN=2,8146000/ADAM9=1}	1.96	1.96	1.00	1.00	Inf	2.00	1.96	1.20	11.00	1.00
{7897068/SKI=1,7912692/HSPB7=1,8067233/PMEPA1=1}	{7981142/CLMN=2,8146000/ADAM9=1}	1.94	1.94	0.83	0.92	6.00	1.83	1.94	1.20	11.00	1.00
{7897068/SKI=1,7959298/TMEM120B=1,8067233/PMEPA1=1}	{7981142/CLMN=2,8146000/ADAM9=1}	1.94	1.94	1.00	1.00	Inf	2.00	1.94	1.20	11.00	1.00
{7959298/TMEM120B=1,7912692/HSPB7=1,8067233/PMEPA1=1}	{7981142/CLMN=2,8146000/ADAM9=1}	1.96	1.96	1.00	1.00	Inf	2.00	1.96	1.20	11.00	0.00
{7897068/SKI=1,7959298/TMEM120B=1,7912692/HSPB7=1,8067233/PMEPA1=1}	{7981142/CLMN=2,8146000/ADAM9=1}	1.96	1.96	1.00	1.00	Inf	2.00	1.96	1.20	11.00	1.00
