# Supplementary material for: eXplainable Artificial Intelligence (XAI) for the identification of biologically relevant gene expression patterns in longitudinal human studies, insights from obesity research
Source: PLoS Comput Biol. 2020 Apr 10;16(4):e1007792. doi: 10.1371/journal.pcbi.1007792 (PMC7176286; doi:10.1371/journal.pcbi.1007792)
Supplement: S3 Table — (PDF) [file pcbi.1007792.s012.pdf]

Supplementary table 3. The complete set of sequential rules that contain at least one of the identified key LHS and RHS loci (from the validation populations).

LHS	RHS	BP	CC	CF	CONF	CONV	LIFT	MF	SP	SUP	TF	Dataset
{7980970/ITPK1=1}	{7927285/FAM35DP|RHEB=1}	1.70	1.70	0.23	0.65	1.30	1.19	1.70	6.00	11.00	0.00	GSE77962_VLCD
{7928872/SNCG=1}	{8101992/SLC39A8=1}	1.90	1.90	0.47	0.80	1.88	1.28	1.90	1.20	12.00	0.00	GSE77962_VLCD
{7928872/SNCG=1}	{8146000/ADAM9=1}	1.71	1.71	0.36	0.73	1.56	1.26	1.71	1.20	11.00	0.00	GSE77962_VLCD
{7959298/TMEM120B=1}	{8146000/ADAM9=1}	1.88	1.88	0.82	0.92	5.42	1.58	1.88	1.20	12.00	0.00	GSE77962_VLCD
{7959298/TMEM120B=1}	{8160297/PLIN2=1}	1.92	1.92	0.69	0.85	3.25	1.69	6.00	1.20	11.00	0.00	GSE77962_VLCD
{7959298/TMEM120B=1}	{7981142/CLMN=2}	1.88	1.88	0.63	0.85	2.71	1.45	1.88	1.20	11.00	0.00	GSE77962_VLCD
{7980970/ITPK1=1}	{8101992/SLC39A8=1}	1.92	1.92	0.22	0.71	1.28	1.13	1.92	6.00	12.00	0.00	GSE77962_VLCD
{7980970/ITPK1=1}	{8141094/PDK4=1}	1.69	1.69	0.15	0.65	1.18	1.11	1.69	6.00	11.00	0.00	GSE77962_VLCD
{7980970/ITPK1=1}	{7935776/=2}	6.00	6.00	0.29	0.65	1.42	1.29	6.00	6.00	11.00	0.00	GSE77962_VLCD
{7980970/ITPK1=1}	{8094679/KLB=2}	1.80	1.80	0.23	0.65	1.30	1.19	1.80	6.00	11.00	0.00	GSE77962_VLCD
{7980970/ITPK1=1}	{8171172/MXRA5=2}	1.98	1.98	0.15	0.65	1.18	1.11	1.98	6.00	11.00	0.00	GSE77962_VLCD
{7999591/ABCC6=1}	{7981142/CLMN=2}	1.91	1.91	0.25	0.69	1.33	1.18	1.91	1.20	11.00	0.00	GSE77962_VLCD
{8034940/NOTCH3=1}	{7981142/CLMN=2}	1.79	1.79	0.63	0.85	2.71	1.45	1.79	6.00	11.00	1.00	GSE77962_VLCD
{8074388/SLC25A1=1}	{8101992/SLC39A8=1}	1.84	1.84	0.43	0.79	1.75	1.26	1.84	1.20	11.00	0.00	GSE77962_VLCD
{8074388/SLC25A1=1}	{8146000/ADAM9=1}	1.83	1.83	0.49	0.79	1.94	1.35	1.83	1.20	11.00	0.00	GSE77962_VLCD
{8131326/SLC29A4=1}	{8101992/SLC39A8=1}	1.88	1.88	0.43	0.79	1.75	1.26	1.88	1.20	11.00	0.00	GSE77962_VLCD
{7995574/HNRNPA1=2}	{8101992/SLC39A8=1}	1.90	1.87	0.59	0.85	2.44	1.35	1.90	1.20	11.00	0.00	GSE77962_VLCD
{8022814/HNRNPA1=2}	{8101992/SLC39A8=1}	1.90	1.87	0.59	0.85	2.44	1.35	1.90	1.20	11.00	0.00	GSE77962_VLCD
{8034313/HNRNPA1=2}	{8101992/SLC39A8=1}	1.90	1.87	0.59	0.85	2.44	1.35	1.90	1.20	11.00	0.00	GSE77962_VLCD
{8038942/ZNF432=2}	{8101992/SLC39A8=1}	1.94	1.94	0.78	0.92	4.50	1.47	1.94	1.20	11.00	0.00	GSE77962_VLCD
{8051622/SRSF7=2}	{8101992/SLC39A8=1}	1.92	1.92	0.29	0.73	1.41	1.17	1.92	6.00	11.00	0.00	GSE77962_VLCD
{8131326/SLC29A4=1}	{8146000/ADAM9=1}	1.89	1.89	0.49	0.79	1.94	1.35	1.89	1.20	11.00	0.00	GSE77962_VLCD
{8140468/GSAP=2}	{7976795/MEG3=2}	6.00	6.00	0.49	0.79	1.94	1.35	6.00	1.20	11.00	0.00	GSE77962_VLCD
{8140468/GSAP=2}	{8171172/MXRA5=2}	1.97	1.97	0.49	0.79	1.94	1.35	1.97	1.20	11.00	0.00	GSE77962_VLCD
{7928872/SNCG=1}	{7932227/NMT2=1}	1.78	1.78	0.39	0.75	1.64	1.27	1.78	1.20	9.00	0.00	GSE77962_LCD
{7928872/SNCG=1}	{8166079/EGFL6=1}	1.87	1.87	0.27	0.83	1.36	1.08	1.87	1.20	10.00	0.00	GSE77962_LCD

{7928872/SNCG=1}	{7928872/SNCG=2}	1.00	1.00	0.67	0.83	3.00	1.67	1.00	1.20	10.00	0.00	GSE77962_LCD
{8034940/NOTCH3=1}	{7932227/NMT2=1}	1.59	1.59	0.13	0.64	1.15	1.09	1.59	6.00	9.00	1.00	GSE77962_LCD
{8061227/SLC24A3=1}	{7932227/NMT2=1}	1.87	1.87	0.13	0.64	1.15	1.09	1.87	1.20	9.00	0.00	GSE77962_LCD
{8061227/SLC24A3=1}	{7945232/ADAMTS15=1}	1.92	1.92	0.29	0.64	1.40	1.29	1.92	1.20	9.00	0.00	GSE77962_LCD
{8172043/SRPX=2}	{7945232/ADAMTS15=1}	1.94	1.94	0.50	0.75	2.00	1.50	6.00	1.20	9.00	0.00	GSE77962_LCD
{7980970/ITPK1=1}	{8032829/PLIN4=2}	6.00	1.90	0.50	0.75	2.00	1.50	6.00	6.00	9.00	0.00	GSE77962_LCD
{8061227/SLC24A3=1}	{8005475/TRIM16|TRIM16L=1}	1.84	1.84	0.37	0.86	1.59	1.11	1.84	1.20	12.00	0.00	GSE77962_LCD
{8032829/PLIN4=1}	{8111864/C6=1}	6.00	1.92	0.48	0.69	1.92	1.69	6.00	6.00	9.00	0.00	GSE77962_LCD
{8032829/PLIN4=1}	{7928872/SNCG=2}	6.00	1.89	0.38	0.69	1.63	1.38	6.00	1.20	9.00	0.00	GSE77962_LCD
{8032829/PLIN4=1}	{8001457/CES1=2}	6.00	1.89	0.44	0.69	1.77	1.52	6.00	6.00	9.00	0.00	GSE77962_LCD
{8032829/PLIN4=1}	{8032829/PLIN4=2}	6.00	1.83	0.54	0.77	2.17	1.54	6.00	1.20	10.00	0.00	GSE77962_LCD
{8034940/NOTCH3=1}	{8166079/EGFL6=1}	1.79	1.79	0.37	0.86	1.59	1.11	1.79	6.00	12.00	1.00	GSE77962_LCD
{8034940/NOTCH3=1}	{8004057/KIF1C=2}	1.76	1.76	0.35	0.64	1.53	1.41	1.76	6.00	9.00	1.00	GSE77962_LCD
{8034940/NOTCH3=1}	{8032829/PLIN4=2}	6.00	1.84	0.29	0.64	1.40	1.29	6.00	6.00	9.00	1.00	GSE77962_LCD
{8061227/SLC24A3=1}	{8137526/INSIG1=1}	1.86	1.86	0.43	0.71	1.75	1.43	1.86	1.20	10.00	0.00	GSE77962_LCD
{8061227/SLC24A3=1}	{7988283/EIF3J-AS1=2}	6.00	6.00	0.21	0.64	1.27	1.18	6.00	1.20	9.00	0.00	GSE77962_LCD
{8061227/SLC24A3=1}	{8032829/PLIN4=2}	6.00	1.94	0.29	0.64	1.40	1.29	6.00	1.20	9.00	0.00	GSE77962_LCD
{8087224/SLC25A20=1}	{8166079/EGFL6=1}	1.96	1.96	0.27	0.83	1.36	1.08	1.96	1.20	10.00	0.00	GSE77962_LCD
{239043_at/ZNF404=1}	{213107_at/TNIK=2}	1.42	1.42	1.00	1.00	Inf	1.44	1.42	1.20	8.00	0.00	GSE103766_WEIGHTREGAIN
{218345_at/TMEM176A=2}	{220459_at/MCM3AP-AS1=2}	6.00	6.00	1.00	1.00	Inf	1.44	6.00	1.20	9.00	0.00	GSE103766_WEIGHTREGAIN
{221919_at/HNRNPA1=2}	{1559261_a_at/PKD1L2=1}	1.92	1.92	1.00	1.00	Inf	1.50	1.92	6.00	4.00	0.00	GSE103766_WEIGHTLOSS
{221919_at/HNRNPA1=2}	{208229_at/FGFR2=1}	1.00	1.00	1.00	1.00	Inf	1.50	1.00	6.00	4.00	0.00	GSE103766_WEIGHTLOSS
{1558755_x_at/ZNF763=2}	{211623_s_at/FBL=2}	1.73	1.73	1.00	1.00	Inf	1.20	1.73	6.00	4.00	0.00	GSE103766_WEIGHTLOSS
{210168_at/C6=2}	{1566780_at/=2}	6.00	6.00	1.00	1.00	Inf	1.50	6.00	6.00	4.00	0.00	GSE103766_WEIGHTLOSS
{210168_at/C6=2}	{221950_at/EMX2=2}	1.72	1.72	1.00	1.00	Inf	1.50	1.72	6.00	4.00	0.00	GSE103766_WEIGHTLOSS
{210168_at/C6=2}	{227410_at/FAM43A=2}	6.00	6.00	1.00	1.00	Inf	1.50	6.00	6.00	4.00	0.00	GSE103766_WEIGHTLOSS
{210168_at/C6=2}	{243244_at/CACNB4=2}	1.83	1.83	1.00	1.00	Inf	1.20	1.83	6.00	4.00	0.00	GSE103766_WEIGHTLOSS
{229634_at/TMEM139=1}	{201129_at/SRSF7=1}	6.00	1.97	1.00	1.00	Inf	1.60	1.97	6.00	5.00	0.00	GSE35411
{201129_at/SRSF7=2}	{201129_at/SRSF7=1}	1.05	1.05	1.00	1.00	Inf	1.60	1.05	6.00	5.00	0.00	GSE35411

{210168_at/C6=2}	{201129_at/SRSF7=1}	1.77	1.77	1.00	1.00	Inf	1.60	1.77	6.00	5.00	0.00	GSE35411
{229634_at/TMEM139=1}	{210168_at/C6=1}	6.00	1.94	1.00	1.00	Inf	1.60	1.94	6.00	5.00	0.00	GSE35411
{201129_at/SRSF7=2}	{210168_at/C6=1}	1.77	1.77	1.00	1.00	Inf	1.60	1.77	6.00	5.00	0.00	GSE35411
{210168_at/C6=2}	{210168_at/C6=1}	1.00	1.00	1.00	1.00	Inf	1.60	1.00	6.00	5.00	0.00	GSE35411
{229634_at/TMEM139=1}	{211673_s_at/MOCS1=1}	6.00	1.98	1.00	1.00	Inf	1.60	1.98	6.00	5.00	0.00	GSE35411
{201129_at/SRSF7=2}	{211673_s_at/MOCS1=1}	1.79	1.79	1.00	1.00	Inf	1.60	1.79	6.00	5.00	0.00	GSE35411
{210168_at/C6=2}	{211673_s_at/MOCS1=1}	1.86	1.86	1.00	1.00	Inf	1.60	1.86	6.00	5.00	0.00	GSE35411
{229634_at/TMEM139=1}	{214455_at/HIST1H2BC=2}	6.00	1.97	1.00	1.00	Inf	1.60	1.97	6.00	5.00	0.00	GSE35411
{229634_at/TMEM139=1}	{215071_s_at/HIST1H2AC=2}	6.00	1.97	1.00	1.00	Inf	1.60	1.97	6.00	5.00	0.00	GSE35411
{229634_at/TMEM139=1}	{230246_at/PLAC9=2}	6.00	1.97	1.00	1.00	Inf	1.33	1.97	1.20	5.00	0.00	GSE35411
{229634_at/TMEM139=1}	{232035_at/HIST1H4H=2}	6.00	1.97	1.00	1.00	Inf	1.60	1.97	1.20	5.00	0.00	GSE35411
{201129_at/SRSF7=2}	{214455_at/HIST1H2BC=2}	1.72	1.72	1.00	1.00	Inf	1.60	1.72	6.00	5.00	0.00	GSE35411
{201129_at/SRSF7=2}	{215071_s_at/HIST1H2AC=2}	1.74	1.74	1.00	1.00	Inf	1.60	1.74	6.00	5.00	0.00	GSE35411
{201129_at/SRSF7=2}	{230246_at/PLAC9=2}	6.00	1.97	1.00	1.00	Inf	1.33	1.97	6.00	5.00	0.00	GSE35411
{201129_at/SRSF7=2}	{232035_at/HIST1H4H=2}	1.51	1.51	1.00	1.00	Inf	1.60	1.51	6.00	5.00	0.00	GSE35411
{204955_at/SRPX=2}	{224735_at/CYB561A3=2}	1.87	1.87	1.00	1.00	Inf	1.60	1.87	1.20	5.00	0.00	GSE35411
{210168_at/C6=2}	{214455_at/HIST1H2BC=2}	1.75	1.75	1.00	1.00	Inf	1.60	1.75	6.00	5.00	0.00	GSE35411
{210168_at/C6=2}	{215071_s_at/HIST1H2AC=2}	1.86	1.86	1.00	1.00	Inf	1.60	1.86	6.00	5.00	0.00	GSE35411
{210168_at/C6=2}	{230246_at/PLAC9=2}	6.00	1.97	1.00	1.00	Inf	1.33	1.97	6.00	5.00	0.00	GSE35411
{210168_at/C6=2}	{232035_at/HIST1H4H=2}	1.65	1.65	1.00	1.00	Inf	1.60	1.65	6.00	5.00	0.00	GSE35411
{7945774/SLC22A18AS=1}	{7900699/CDC20=1}	1.97	1.97	1.00	1.00	Inf	1.13	1.97	6.00	6.00	0.00	GSE70529
{8140478/GSAP=2}	{7900699/CDC20=1}	1.56	1.56	1.00	1.00	Inf	1.13	1.56	6.00	6.00	0.00	GSE70529
{8140478/GSAP=2}	{7935572/MIR1287|PYROXD2=1}	1.87	1.90	1.00	1.00	Inf	1.29	1.95	1.20	6.00	0.00	GSE70529
{7945774/SLC22A18AS=1}	{7966150/SSH1=1}	1.97	1.97	1.00	1.00	Inf	1.13	1.97	6.00	6.00	0.00	GSE70529
{7945774/SLC22A18AS=1}	{7966829/WSB2=1}	1.98	1.98	0.00	1.00	1.00	1.00	6.00	1.20	6.00	0.00	GSE70529
{7945774/SLC22A18AS=1}	{8107321/EPB41L4A-AS1=2}	6.00	6.00	0.00	1.00	1.00	1.00	6.00	1.20	6.00	0.00	GSE70529
{7945774/SLC22A18AS=1}	{8140356/HIP1=2}	1.97	1.97	1.00	1.00	Inf	1.13	1.97	1.20	6.00	0.00	GSE70529
{8140478/GSAP=2}	{7966150/SSH1=1}	1.66	1.66	1.00	1.00	Inf	1.13	1.66	6.00	6.00	0.00	GSE70529
{8140478/GSAP=2}	{7966829/WSB2=1}	1.80	1.80	0.00	1.00	1.00	1.00	6.00	1.20	6.00	0.00	GSE70529

{8140478/GSAP=2}	{8114455/HSPA9=1}	1.77	1.77	1.00	1.00	Inf	1.29	1.77	1.20	6.00	0.00	GSE70529
{8140478/GSAP=2}	{8114805/FGF1=1}	1.56	1.56	1.00	1.00	Inf	1.13	1.56	1.20	6.00	0.00	GSE70529
{8140478/GSAP=2}	{8135909/LEP=1}	1.66	1.66	1.00	1.00	Inf	1.29	6.00	1.20	6.00	0.00	GSE70529
{8140478/GSAP=2}	{7949857/NUDT8=2}	6.00	6.00	1.00	1.00	Inf	1.50	1.98	1.20	6.00	0.00	GSE70529
{8140478/GSAP=2}	{8107321/EPB41L4A-AS1=2}	6.00	6.00	0.00	1.00	1.00	1.00	6.00	1.20	6.00	0.00	GSE70529
{218345_at/TMEM176A=2}	{212266_s_at/SRSF5=2}	6.00	1.98	1.00	1.00	Inf	1.44	6.00	6.00	8.00	0.00	GSE103766_WEIGHTREGAIN
