# Supplementary material for: eXplainable Artificial Intelligence (XAI) for the identification of biologically relevant gene expression patterns in longitudinal human studies, insights from obesity research
Source: PLoS Comput Biol. 2020 Apr 10;16(4):e1007792. doi: 10.1371/journal.pcbi.1007792 (PMC7176286; doi:10.1371/journal.pcbi.1007792)
Supplement: Attachment — Submitted filename: 2019_PCOMPBIOL_D_19_01444_Review.pdf [file pcbi.1007792.s013.pdf]

eXplainable Artificial Intelligence (XAI) for the identification of biologically relevant gene expression networks in longitudinal human studies
PLoS Computational Biology Reviewer Report
September 30, 2019

Article summary

A recent trend in computational biology has been to develop algorithmic approaches for extracting networks from gene expression data (GED). Some of the methods involved—namely, some of the machine learning (ML) methods—in this process are powerful but can be challenging for humans to interpret. The current work describes a pipeline for extracting co-expression networks from data, and the authors apply this algorithm to 83 gene expression datasets from six experimental groups of humans (four independent cohorts, associated with obesity). By reconstructing co-expression networks, the authors were able to validate certain connections already found in the literature and verified through experts. This pipeline is associated with a public tool that attempts to give practitioners the ability to construct such networks themselves.

Review summary

I appreciate the intuition behind this research, and I agree with the authors' claim that it represents an advance both in the methodology for modeling temporal -omics data but also in possible insights that may arise from such modeling. However, in its current form, it is not ready to be published in *PLoS Computational Biology*, and I believe it needs to undergo substantial improvements in order to be published at all. This is not to say that the article lacks merit, nor that its core ideas are not worth pursuing. Rather, by reorganizing the paper and refocusing it around the results derived from the methods introduced, I think the paper would be more compelling to a broader computational biology audience.

Some of the comments in this review may come across as harsh or blunt, which is not my intention—I earnestly would like to help improve this research however I can.

I. General comments and requests

1. Overall structure of the article

1. Throughout, I found myself wondering if this is a "methods paper" or a "results paper" or a "case study" etc. That is, I had a difficult time understanding what the authors believed their main contribution(s) were.
   - If the authors believe that the main contribution is a methodological one, then I would like to see more comparison with other state-of-the-art tools for network reconstruction. There are many, and I would question whether the eXplainable approach described in this paper is the only one that is human-interpretable.
   - If the authors believe that their main contributions are about obesity and human genomics, then that should have featured much more prominently.
     - "An exhaustive study of all the results from the case of study is needed otherwise to understand the concrete molecular patterns underlying WL-induced responses in obesity." If the authors were able to do this, it would be a much stronger contribution.
   - If the authors believe that their main contributions are providing an open-source tool for practitioners to extract networks from their gene expression data, then the paper should have focused on that.
2. All of the above frames are worthwhile, but as it stands now, the article appears to try to accommodate each of them simultaneously. The effect is that the paper is quite difficult to read in some parts, and the results that the authors present can often seem unrelated to the motivating questions behind this work.

2. Especially with gene network reconstruction, it is important to compare against a benchmark

1. The authors make several points about how useful their approach is and how important it is to have eXplainable methods in machine learning. I would have like to see a more in-depth comparison of different methods for reconstructing these gene co-expression networks.
2. If the authors opted not to include alternative methods in their analyses, they need a better justification of why such methods are not suited for the task at hand. Otherwise, I am left wondering about the performance of other methods for inferring connections between nodes[1–3].

3. Network visualizations are not the same as network analysis

1. I encourage the authors to be careful when treating network visualizations as if they are core results. The authors write, "Data visualization techniques have been widely employed in data science applications given their ability to transform raw data into useful knowledge," citing a book from 2001 that is not related to the types of visualizations that the authors' tool offers.

2. More generally, if the visualization tool is a primary contribution of this work, I further do not think the paper is suited for *PLoS Computational Biology*. Visualization tools are surely meaningful contributions in science, but I suspect they are better-suited for different venues.

II. Specific comments and requests

1. In general, every figure is very challenging to follow.

   - Figure 1: This figure is hardly referred to in the text, the figure caption does not justify the rationale behind the point scheme. Visually, I have a very tough time reading the thin, faint white text on the colorful background. The caption does not introduce the figure in a meaningful sense.
   - Figure 2 and Figure 3: The legends on the right side of these figured need to be improved. They also appear to be the exact same figure as Supplemental Figure 4, and I do not understand why. I do not understand why there is a pie chart in the legend. However, given that there is a pie chart, I do not understand why it is made up of five equivalently-sized portions, when the values are in increments of {0.25, 0.25, 0.25, 0.25, 1.0}. The colormap extends to a color that is almost entirely white, making several network edges almost invisible. The font size makes it very challenging to read. The node labels do not offer anything to the reader who is unfamiliar with what those labels mean.
   - Figure 4: The "x" in many of the cells makes the figure challenging to interpret.
   - Figure 5: While visually appealing, this figure could easily be summarized in a couple of sentences. The method has uncovered a couple of meaningful relationships in the data: I would find it more compelling to have those explored more thoroughly in the text rather than in this figure.

2. I could not tell whether this sentence meant that experts *did* or *did not* evaluate the results from this study. "In order to assess the biological utility of our gene networking strategy, obesity-field experts evaluated all extracted rules making use of the computed biological quality measures and the graphical representations." If the authors showed their methods and results to experts and used their insights to further inform or validate their methods, that procedure should have been much more thoroughly described.

3. The following sentences are in the results section, and I believe they are intended to be interpreted as results from this study: "In general, extracted rules presented good values for all computed quality measures, which indicates a good performance of the algorithm during the gene association mining process." and "Of note, top sequential rules also presented good rates in the new proposed biological quality measures." Describing values or rates as good is not informative unless the reader has a benchmark to compare to, and even then it should be avoided. As such, I do not know how to interpret much of the results in the Results section.

4. While the authors indeed mention that "Although extracted sequential rules may seem to indicate causal relations among genes, it is important to note that, in the majority of the cases, they just represent indirect relationships between items," I would have liked them to dive more into what it means for two nodes to have a directed connection in their reconstructed networks. The authors say that the connections "might be illustrating how different biological processes take place in AT one after the other, and how there may exist some functional connections between them," but this still does not address the basic question of what a network edge *means*.

III. Minor comments and non-urgent requests

1. I would earnestly like to offer substantive, line-by-line feedback on this article, but the format often made it challenging. In the future, small things like page numbers and line numbers can go a long way towards helping reviewers offer substantive feedback.

- I strongly recommend referring to the *PLoS Computational Biology* manuscript formatting page hyperlinked here prior to submitting this article.
- If the authors write their papers using LaTeX, *PLoS Computational Biology* also has a publicly-available template, hyperlinked here.

2. Table 1 is very disorganized and makes the descriptions inside it hard to follow.
3. There are so many acronyms and abbreviations, and it is often not clear if they refer to the authors' own methodology or if they are common in the machine learning literature.
4. While the graphical abstract is visually appealing, I struggle to interpret most of it. The icons are sleak, but I think that simpler figure that focuses on the specific contributions in this work is much more compelling.

References

1. Abdul Razak, F. & Jensen, H. J. Quantifying 'causality' in complex systems: Understanding transfer entropy. *PLoS ONE* **9**, 1–14, DOI: 10.1371/journal.pone.0099462 (2014).

2. Zhou, D., Xiao, Y., Zhang, Y., Xu, Z. & Cai, D. Granger causality network reconstruction of conductance-based integrate-and-fire neuronal systems. *PLoS ONE* **9**, DOI: 10.1371/journal.pone.0087636 (2014).

3. Runge, J. Causal network reconstruction from time series: From theoretical assumptions to practical estimation. *Chaos* **28**, DOI: 10.1063/1.5025050 (2018).
