# Supplementary material for: eXplainable Artificial Intelligence (XAI) for the identification of biologically relevant gene expression patterns in longitudinal human studies, insights from obesity research
Source: PLoS Comput Biol. 2020 Apr 10;16(4):e1007792. doi: 10.1371/journal.pcbi.1007792 (PMC7176286; doi:10.1371/journal.pcbi.1007792)
Supplement: Attachment — Submitted filename: RESPONSE TO REVIEWERS_2019_PCOMPBIOL_D1901444_16_12_19.docx [file pcbi.1007792.s014.docx]

Granada, 13th of December 2019.

Dear editor and reviewers,

This is an updated version of the paper **PCOMPBIOL-D-19-01444**, where we include the description of changes and comments on the referees' questions.

**Response to the Reviewers to the Report on PCOMPBIOL-D-19-01444**

*Title of the paper: eXplainable Artificial Intelligence (XAI) for the identification of biologically relevant gene expression patterns in longitudinal human studies: Insights from obesity research*

*(A. Anguita-Ruiz, A. Segura-Delgado, R. Alcalá, C.M. Aguilera & J. Alcalá-Fdez.)*

First, we would like to deeply thank the editor and the reviewers for the revision of our manuscript, which included a range of thoughtful and constructive comments. We have taken into account all the comments as well as solved most of reported concerns. As a result, the manuscript has experimented a full reorganization and has been through extensive changes in the majority of its sections. Now, we think the main core ideas of our proposal are clearer and that the general quality of the paper has considerably increased.

In general, all specific requests from reviewers have been included throughout our manuscript highlighted in yellow and under the “track-changes” option in Microsoft Word. Furthermore, an itemized point-by-point response to each reviewer’s comment is presented below.

Besides addressing each reviewer concern, following editor recommendations, we have been further working in our manuscript to improve English and flow, to update literature as well as to adapt the work-format to the PLOSCOMPBIOL guidelines for publication.

**Important note: Page and line numbers in this document are referred to the clean version of the manuscript.**

Yours faithfully,

Anguita-Ruiz Augusto on behalf of the authors.

Response to Reviewer 1 Comments

**RV: Reviewer**

**AA: Author Answers**

**RV:**

**eXplainable Artificial Intelligence (XAI) for the identification of biologically relevant gene expression networks in longitudinal human studies**

**PLoS Computational Biology Reviewer Report**

**September 30, 2019**

**Article summary**

A recent trend in computational biology has been to develop algorithmic approaches for extracting networks from gene expression data (GED). Some of the methods involved—namely, some of the machine learning (ML) methods—in this process are powerful but can be challenging for humans to interpret. The current work describes a pipeline for extracting co-expression networks from data, and the authors apply this algorithm to 83 gene expression datasets from six experimental groups of humans (four independent cohorts, associated with obesity). By reconstructing co-expression networks, the authors were able to validate certain connections already found in the literature and verified through experts. This pipeline is associated with a public tool that attempts to give practitioners the ability to construct such networks themselves.

**Review summary**

I appreciate the intuition behind this research, and I agree with the authors’ claim that it represents an advance both in the methodology for modeling temporal -omics data but also in possible insights that may arise from such modeling. However, in its current form, it is not ready to be published in PLoS Computational Biology, and I believe it needs to undergo substantial improvements in order to be published at all. This is not to say that the article lacks merit, nor that its core ideas are not worth pursuing. Rather, by reorganizing the paper and refocusing it around the results derived from the methods introduced, I think the paper would be more compelling to a broader computational biology audience. Some of the comments in this review may come across as harsh or blunt, which is not my intention—I earnestly would like to help improve this research however I can.

**I. General comments and requests**

**1. Overall structure of the article**

1. Throughout, I found myself wondering if this is a “methods paper” or a “results paper” or a “case study” etc. That is, I had a difficult time understanding what the authors believed their main contribution(s) were.

- If the authors believe that the main contribution is a methodological one, then I would like to see more comparison with other state-of-the-art tools for network reconstruction. There are many, and I would question whether the eXplainable approach described in this paper is the only one that is human-interpretable.
- If the authors believe that their main contributions are about obesity and human genomics, then that should have featured much more prominently.

“An exhaustive study of all the results from the case of study is needed otherwise to understand the concrete molecular patterns underlying WL-induced responses in obesity.” If the authors were able to do this, it would be a much stronger contribution.

- If the authors believe that their main contributions are providing an open-source tool for practitioners to extract networks from their gene expression data, then the paper should have focused on that.

2. All of the above frames are worthwhile, but as it stands now, the article appears to try to accommodate each of them simultaneously. The effect is that the paper is quite difficult to read in some parts, and the results that the authors present can often seem unrelated to the motivating questions behind this work.

**2. Especially with gene network reconstruction, it is important to compare against a benchmark**

1. The authors make several points about how useful their approach is and how important it is to have eXplainable methods in machine learning. I would have like to see a more in-depth comparison of different methods for reconstructing these gene co-expression networks.
2. If the authors opted not to include alternative methods in their analyses, they need a better justification of why such methods are not suited for the task at hand. Otherwise, I am left wondering about the performance of other methods for inferring connections between nodes1–3.

**References**

1. Abdul Razak, F. & Jensen, H. J. Quantifying ’causality’ in complex systems: Understanding transfer entropy. PLoS ONE 9, 1–14, DOI: 10.1371/journal.pone.0099462 (2014).
2. Zhou, D., Xiao, Y., Zhang, Y., Xu, Z. & Cai, D. Granger causality network reconstruction of conductance-based integrate-and-fire neuronal systems. PLoS ONE 9, DOI: 10.1371/journal.pone.0087636 (2014).
3. Runge, J. Causal network reconstruction from time series: From theoretical assumptions to practical estimation. Chaos 28, DOI: 10.1063/1.5025050 (2018).

**AA:**

As we have previously announced, we would like to earnestly thank you for such a quality revision and for arising substantial issues in our work. We have accomplished all your proposed suggestions as well as answered each expressed concern. We hope you find our revised manuscript and responses satisfactory.

First, we would like to express that we agree in the fact that our manuscript, in its past form, presented a disorganized structure that could sometimes be leading our main contributions get blurred through by the different parts of the paper. For example, we have noticed that while in the **Introduction** section we made a great focus on highlighting the strengths of our paper as a methodology proposal, **Results and Discussion** sections were otherwise mainly focused on describing and interpreting obesity results without connecting them with our pipeline approach. Moreover, in our **Methods** section there was **not** a clear separation between the parts corresponding to the pipeline description and the parts corresponding to the description of the application of our pipeline to the example datasets. On the contrary, we think it would has been expected:

- To get deepen into **the interpretability of our approach**, highlighting its **differences over other gene-networking approaches** (such as co-expression) and discussing our decision of **why** **not comparing *vs.* a benchmark**.
- **Improving** the **description of the technical parts of the methodology**, splitting them from the description of study datasets and the application of the pipeline to the obesity problem.
- Discussing about what were the **motivations for designing each part of the pipeline**.
- To get deepen into critical methodologic issues such as the **performance, strengths and weaknesses of each part of the pipeline**.
- Discussing in more detail whether the **method behave as expected in presented cases of studies**, interconnecting all study results with each methodology resource responsible for that finding.

Only in this way, it would has been cleared that **our value proposal** was to present a **new pipeline** to face a **research problem that had not been previously addressed** by available co-expression methods (as we explain below). The research problem was the issue of **sequential** causal relationships between **gene expression changes** in humans. In order to face this problem, our pipeline include **new and original steps** as well as **already existent methods**. The whole pipeline is further associated with a **public tool** and could be summarized in the next steps:

•	Feature-selection: The initial number of probes is reduced to those differentially expressed by time interval and experimental condition. This way, we simplify the experimental problem and reduce the search space, further favoring a better performance of the algorithm.

•	Data discretization: We propose a new discretization approach for the conversion of continuous gene expression values into discrete categories representing temporal changes in gene expression. Based on signal log ratios by gene and time interval, this discretization strategy maps data from a vast spectrum of numeric gene expression values into three discrete categories. Therefore, it can be viewed as a secondary data dimensionality reduction technique in favor of model explicability.

•	Knowledge-extraction: We apply the sequential rule mining algorithm CMRules to the discretized dataset and generate sequential rules from it with the form of [gene A↑, gene B↓] → (time delay) [gene C↑, gene D↑, gene E↑]. Each rule is assessed in terms of quality and robustness by means of five interestingness or quality metrics.

•	Functional validation: We propose the integration of output gene rules along with external biological resources such as functional annotation and gene regulation databases. Three well-known and reliable biological resources (GO, KEGG and TRRUST databases) are consulted in order to compute five new biological quality measures by-rule. Through this strategy, each interaction result is biologically pruned and placed within the context of those molecular systems that commonly underlie gene-gene interactions in humans (e.g. Transcription factor (TF)-target gene regulatory relationships).

•	Exploration of results: We propose data visualization for the joint representation of gene patterns and all accessed biological information. By means of hierarchical edge bundling visualization methods, we concentrate a lot of information in a single shot and facilitate the exploration of a finite set of genes composing a good quality network.

With this work, we thus pretended to share a pipeline **serving as a link between high throughput omics analytics** approaches and **functional validation studies**.

In order to amend problems reported by the reviewer and highlight mentioned **methodological** virtues of our work, the manuscript has been through by changes in all its sections and has experienced a strong structure reorganization.

Although the best way to recognize introduced changes in the paper is to perform a whole revision of the work, we next highlight some of the main important changes conducted regarding previously-mentioned issues, directly referring to the parts of the manuscript where they have been introduced:

- Discussion of the **interpretability of the approach**, highlighting its **differences over other gene-networking approaches (such as co-expression)** and **discussing our decision of not comparing *vs*. a benchmark**:

In gene networking, one of the main concerns of biologists has been how to translate inferred networks into particular hypotheses that can be tested with real-life experiments. On this sense, sequential rule mining (SRM) algorithms could be used, obtaining sequential rules with the next form; [gene A↑, gene B↓] → (time delay) [gene C↑, gene D↑, gene E↑], which represents that the upregulation of gene A and the significant repression of gene B are followed by (or cause) a significant upregulation of genes C, D and E after a given time delay. These results, in the form of individual rules, are perfectly interpretable by biologists and allow the generation of robust gene-gene interaction hypotheses (since SRM counts on specific quality metrics by rule). Moreover, they open up the door to new ways of functional validation of results by means of accessing and consulting current biological knowledge in databases. Despite all these utilities, there has been only one adaptation of SRM methods to temporal microarray data (consisting of an Ad-hoc application for in vitro time-series GED in Saccharomyces cerevisiae).

**Co-expression approaches have become otherwise the gold-standard** when studying a microarray experiment from a systems biology perspective. In the case of time course genomics experiments, most of conducted co-expression approaches have been based in clustering analyses or unsupervised learning without class labels. These temporal co-expression approaches are generally based on similarity or correlation or distance measures for the identification of groups of genes with ‘similar’ temporal patterns of expression, and reveal hidden patterns in the original data by transforming raw temporal data into logically structured, clustered, and interconnected graphs. **Co-expression graphs** can be visualized with nodes representing genes, and with edges indicating interactions, and have helped to understand how genes interact each other within the context of an integrated and global biological network. Nevertheless, despite the widespread use of these approaches, there are some **drawbacks and limitations** remaining for their application in the inference of causal gene-gene relationships [1–6]:

- Co-expression networks are good to study the general interactome of an organism (free-scale network topology), but their results make hard to infer particular details such as the causal direction or the importance of each individual interaction within the whole network. Thus, they may hinder the translation of inferred networks into particular hypotheses that can be tested in wet-lab experiments. On the contrary, SRM results (in the form of individual rules for each interaction) would allow a concrete quality evaluation for each relationship and an easy biological interpretability of findings, which is crucial to demonstrate that a gene network is functionally meaningful, and not just biostatistical fluke.

- Co-expression is a very strict assumption for the extraction of gene-gene interactions from time course data. That is to say, co-expression networks with temporal GED generally do not include the time order information in graphs, and they are not capable of detecting positive, negative and time-lagged gene correlations at the same time. However, in living systems, gene regulations can be positive or negative possibly with time lags, and may also not span all conditions or time points. For example, some of the target genes could have a negative feedback loop and could block their own expression, which could explain fast transient dynamic changes, while other target genes could have a positive feedback loop and therefore maintain gene expression longer. Additional regulation could happen after a longer time or very fast without protein translation, i.e. by the action of functional large non-coding RNAs. All these kind of phenomena, which are missed by most of co-expression approaches, could be captured by an appropriate SRM approach.

Given all these concerns and the interesting properties of SRM methods (e.g. existence of statistical quality measures by rule, possibility of functional validation by interaction, inclusion of causality or sequential order information, discovery of complex temporal regulatory phenomena...), **SRM approaches are presented as an alternative** of great interest and interpretability against temporal co-expression clustering methods when **inferring gene-gene temporal relations**.

Unfortunately, as far as we concern, the work of Nam et al. (2009) in *Sacharomyces cerevisiae* is the only SRM approach developed to the moment and it constitutes no more than an Ad-hoc application for in vitro experiments (whose extension to in vivo GED would elicits challenges that could not be solved with simple algorithm modifications). The application of these methods to human temporal gene profiles otherwise would greatly expand our knowledge for complex biological processes, with a special interest for long-term interventions (such as clinical trials), in which identified gene-gene regulatory interactions could reveal promising and new therapeutic targets.

For this reason, our value proposal was to present a new **SRM pipeline** (associated with a public tool) **to give practitioners and biologists** the ability to:

-	Infer **comprehensible gene-gene temporal relationships** in long-term human intervention studies.

-	**Interpret, investigate and validate** (at the **biological level**) particular hypotheses of **gene-gene interactions**, extracting from them relationships that could be further investigated at the lab with real life experiments.

The strategy presented in our work (with special relevance of the adopted ML algorithm, and the functional validation and graphical representation of results) constitute a value proposal whose main objective was to increase model eXplainability and to help biologists understanding extracted gene interactions. In this way, we pretended to move away from the black box concept that is usually adopted in most of the current artificial intelligence (AI) omics applications, and to provide researchers with a great power to discern between random and causal gene relationships.

Since our method is the **first application of a rule-based SRM** strategy for the extraction of **gene interactions in longitudinal human *in vivo* experiments**, there is not currently a SRM **benchmark** tool that we could use to compare the performance of our pipeline with. At least, not without implementing not trivial algorithm modifications in such comparison method in order to obtain comparable results, thus involving developing another new proposal. In spite of it, the rule extraction step that we are implementing here is based on the CMRules algorithm, and therefore this step itself is not novel, but it has been previously validated as an efficient tool against other ML methods for the extraction of sequential rules in different kind of datasets and situations.

From the biomedical perspective the challenge issue when inferring gene networks is their reliability for avoiding **false discovery** as well as their **reproducibility** across different patient cohorts. For this reason, we decided to validate our approach in two alternative ways: 1) First, we applied our methodology to an example dataset and give the derived results to a group of field-experts in order them to evaluate the usability of inferred networks for the generation of particular gene-gene interaction hypotheses; and 2) We repeated the application of the full pipeline to three additional datasets, following the same experimental design than the discovery sample, and mined results looking for replication patterns across studies.

Full details for the evaluation guidelines committed by field-experts during the interpretation of model results have been addressed in a particular method section.

All this information has been included in our paper and can be found in:

**(Pages 5-7,9-11)** Lines 125-191,235-288 **Introduction** Section.

- **Description of the technical parts of the methodology**, splitting them from the description of study datasets.

**Pure methodological and technical details of our approach** have been separated from the **description of the application of our pipeline to example datasets**. For that purpose, the whole **method section has been rewritten**, illustrating how our method could be applied to **a hypothetical gene expression dataset** (not focused in the obesity field). All this information has been included in our paper in:

**(Pages 11-23)** Lines 289-640 **Method** Section.

Otherwise, the technical particularities regarding the **application of the pipeline to example datasets** have been separately gathered in:

**(Pages 23-26)** Lines 643-719 **Method** Section.

- Discussion about what were **the motivations for designing each part of the pipeline**.

Each part of the pipeline was intended with one particular goal for the final inference of gene-gene temporal patterns. For this reason, the main motivations for each stage (**feature-selection** step, **discretization** approach, **type of SRM algorithm** and **functional validation** stage) have been highlighted in several parts of the paper. Please read:

**(Pages 7-10)** Lines 194-273 **Introduction** Section.

**(Pages 12-22)** Lines 322-343,365-371,425-492,496-506,514-524,528-552,590-599 **Method** Section.

**(Pages 33-34)** Lines 921-973 **Discussion** Section.

- Get into methodologic issues such as the **performance, strengths and weaknesses** of each part of the pipeline.

**(Pages 17-22)** Lines 476-489,496-506,590-599,609-620 **Method** Section.

- Discussion in more detail whether the **method behave as expected in presented cases of studies**, interconnecting all study results with each methodology resource responsible for that finding.

**(Pages 27-28, 30-31)** Lines 741-770,845-887 **Results** Section.

**(Pages 36-37)** Lines 996-1009 **Discussion** Section.

- Future directions.

**(Pages 36-37)** Lines 1007-1039 **Discussion** Section.

We have also read the references proposed by the reviewer and have encountered them as interesting interpretable methods for inferring gene-gene interactions. Nevertheless, again their application to the field of *in vivo* human experiments would involve significant algorithm extensions to generate new methods for comparison to our approach (understanding these modifications as not minor changes of presented methods). Moreover, we have not found any related application of these methods to the case of human long-term interventions with few temporal records available (we only found an example of application for *in vitro* time series with up to 20 time points). In spite of it, we have included them in the discussion of our work as interesting alternatives asserting with the core ideas of XAI revolution:

**(Page 32)** Lines 897-899 **Discussion** Section.

References:

1.	Weiss JN, Karma A, MacLellan WR, Deng M, Rau CD, Rees CM, et al. “Good Enough Solutions” and the Genetics of Complex Diseases. Circ Res. 2012;111: 493–504. doi:10.1161/CIRCRESAHA.112.269084

2.	Liang Y, Kelemen A. Computational dynamic approaches for temporal omics data with applications to systems medicine. BioData Min. 2017;10: 1–20. doi:10.1186/s13040-017-0140-x

3.	Lee W-P, Tzou W-S. Computational methods for discovering gene networks from expression data. Brief Bioinform. 2009;10: 408–23. doi:10.1093/bib/bbp028

4.	Albrecht M, Stichel D, Müller B, Merkle R, Sticht C, Gretz N, et al. TTCA: An R package for the identification of differentially expressed genes in time course microarray data. BMC Bioinformatics. 2017;18: 1–11. doi:10.1186/s12859-016-1440-8

5.	Li J, Lai Y, Zhang C, Zhang Q. TGCnA: temporal gene coexpression network analysis using a low-rank plus sparse framework. J Appl Stat. 2019. doi:10.1080/02664763.2019.1667311

6.	Langfelder P, Horvath S. WGCNA: An R package for weighted correlation network analysis. BMC Bioinformatics. 2008;9. doi:10.1186/1471-2105-9-559

**RV:**

**3. Network visualizations are not the same as network analysis**

1. I encourage the authors to be careful when treating network visualizations as if they are core results. The authors write, “Data visualization techniques have been widely employed in data science applications given their ability to transform raw data into useful knowledge,” citing a book from 2001 that is not related to the types of visualizations that the authors’ tool offers.
2. More generally, if the visualization tool is a primary contribution of this work, I further do not think the paper is suited for PLoS Computational Biology. Visualization tools are surely meaningful contributions in science, but I suspect they are better-suited for different venues.

**AA:**

Thank you very much for your observation. We are sorry that this idea could arise from our manuscript and we have worked to solve the issue with some modifications in the paper.

As we have presented earlier, our visualizations were intended as a part of the pipeline in order to increase model eXplainability (which is far from being an analysis tool for generating networks). The main reason motivating its inclusion in our approach was to obtain a visual representation of the results from a global perspective, which may be somewhat complicated despite the interpretability of each individual rule. Particularly, we though that the evaluation of output rules including their biological quality information would greatly benefit from a visual group approach, where all the rules could be studied together. Thus, we decided to incorporate our visualization tool as an additional stage for the functional validation of the results. We chose a type of plots whose greatest virtue lays in their ability to concentrate different types of information in single shot, which would allow practitioners an easy identification of the top and more coherent rules from the both the technical and biological points of view. On this matter, our visualization tool it is not intended as a network analysis tool (for generating networks from raw data) but only an additional value proposal in favor of model explicability and interpretability.

From the analyzed datasets, computed biological quality measures and the visualization tool demonstrated utility for the biological interpretation of results and the transference of large gene patterns to the expert eye. Thanks to them, field-experts were able to identify several rules corresponding to known biological relationships among genes (e.g. transcription factor-target gene temporal relations or target genes concentrating many connections in the network).

On the other hand, although our CMRules algorithm does not strictly output a network-format like result such as co-expression approaches do, when one visualizes all sequential rules at a single shot, It is evidenced how SRM interestingly keep the scale-free network topology for inferred interactions. This network topology, which is also evidenced by co-expression approaches results, it is in tune with the concept of “good enough solutions” that seems to rule most biological systems and it consists on the existence of a few nodes with many connections (”hubs”) and many nodes with few connections. This, again, demonstrates the suitability of our SRM approach alongside the visualization tool for the modeling genetic interactions in humans.

All this new information has been included in our paper in:

**(Pages 34 and 35)** Lines 973-995 **Discussion** Section.

Furthermore, we have also changed the method section where we introduce our visualization tool in order to include more appropriate references, in which visualization tools are intended to explore already generated knowledge instead of obtain new insights from raw data.

**(Pages 22 and 23)** Lines 621-640 **Method** Section.

Since it could led to confusion, we have also been more rigorous when using the term “networks” and now we only employ it only for referring to the global representations of extracted patterns. In the rest of the cases (e.g. when referring to our method results), the word “network/s” has been replaced by the term “pattern/s” whenever this second one was more appropriate.

**RV:**

**II. Specific comments and requests**

1. In general, every figure is very challenging to follow.

- Figure 1: This figure is hardly referred to in the text, the figure caption does not justify the rationale behind the point scheme. Visually, I have a very tough time reading the thin, faint white text on the colorful background. The caption does not introduce the figure in a meaningful sense.
- Figure 2 and Figure 3: The legends on the right side of these figured need to be improved. They also appear to be the exact same figure as Supplemental Figure 4, and I do not understand why. I do not understand why there is a pie chart in the legend. However, given that there is a pie chart, I do not understand why it is made up of five equivalently-sized portions, when the values are in increments of {0.25, 0.25, 0.25,0.25, 1.0}. The colormap extends to a color that is almost entirely white, making several network edges almost invisible. The font size makes it very challenging to read. The node labels do not offer anything to the reader who is unfamiliar with what those labels mean.
- Figure 4: The "x" in many of the cells makes the figure challenging to interpret.
- Figure 5: While visually appealing, this figure could easily be summarized in a couple of sentences. The method has uncovered a couple of meaningful relationships in the data: I would find it more compelling to have those explored more thoroughly in the text rather than in this figure.

**AA:**

Thank you for the comments.

Regarding figure 1, we tried to represent it in black and white format, notwithstanding, the need for representing with colors the matches in GO-terms made us impossible to keep the change as definitive. Alternatively, we have reduced the level of intensity for background colors and now the white text can be read in a nicer way. We have also added a detailed description and introduction for the figure in itscaption. We hope you find it appropriate.

***“Fig 1.*** *Assignable categories and ranking scores for a rule in the biological measures “BP”, “MF”, “CC” and “SP”. First, a rule will be assigned to a particular category from the bottom category 5 to the top category 1. This assignation will be conducted according to the type of matches encountered for a rule in its annotated terms as is described in the figure. Once a rule is designated to a particular category, a score will be computed for the rule taking into consideration all type of matches encountered for the rule. Each match is weighted with a number of points as illustrated in the figure. The final score for a rule is computed as detailed in the method section.”*

**(Page 44)** Lines 1243-1249 **Table and Figure Legends** Section.

Regarding figure 2 and 3 (now figures 3 and 4), we have improved the legends for each figure following reviewer recommendations. The range 5-6 refers to a category where rules that present no GO-terms matches can be found. These rules are no interesting from a biological point of view. Since the main goal of our visualization tool was to highlight biologically meaningful rules, our purpose was to keep these rules in the background in a very light color. We have also added explanation to figure captions in order to detail what represent the format of node labels.

**(Page 45)** Lines 1259-1262 **Table and Figure Legends** Section.

For figure 4 (now figure 5), we have added explanation into the meaning of the figure and employed “x” symbols.

*“****Fig 5.*** *Correlation between traditional quality metrics and biological quality measures by rule in the sequential rules discovered from the whole GSE77962 dataset (LCD and VLCD groups). R^2^ values quantify the level of correlation for each pair of measures while the level of statistical significance (adjusted by Bonferroni multiple test correction) is evidenced with an X for P-values > 0.05 and nothing for P-values < 0.05.”*

**(Page 44)** Lines 1250-1254 **Table and Figure Legends** Section.

Finally, with regard to figure 5, we have improved the clarity of these ideas in the result section and have moved the figure 5 into the supplementary material (now referred as Sup Fig 9).

**(Page 31)** Lines 861-887 **Results** Section.

**RV:**

2. I could not tell whether this sentence meant that experts did or did not evaluate the results from this study. “In order to assess the biological utility of our gene networking strategy, obesity-field experts evaluated all extracted rules making use of the computed biological quality measures and the graphical representations.” If the authors showed their methods and results to experts and used their insights to further inform or validate their methods, that procedure should have been much more thoroughly described.

**AA:**

Given the absence of a gold-standard which to compare with our approach, and taking into account that the important thing when inferring genetic networks in the biological field is to validate results in independent cohorts, in this work we decided to validate our proposal through its direct application to different cases of study. For that purpose, we applied our methodology to example datasets and **give the derived results to a group of field-experts** in order them to evaluate the usability of inferred networks for the generation of particular gene-gene interaction hypotheses. Field-experts were selected from the research group “CB12/03/30038”, belonging to the Spanish research network CIBEROBN (Physiopathology of Obesity and Nutrition), Institute of Health Carlos III (ISCIII), Madrid, Spain.

On this matter, field-experts received sequential rules results in the form of both tables and figures with traditional quality metrics and biological quality measurements included. The evaluation and interpretation of sequential rules and graphs by field-experts was committed following a few foundations: 1) Rules were ordered according to SRM quality metrics and biological quality measures; 2) Rules with very low values for SRM quality metrics were removed according to the reference values presented in the first method subsection. For this prune, the concept of very strong association rules was taken into account; 3) Correlation analyses were conducted between quality metrics and biological quality measures for remaining rules in order to evaluate the ability of CMRules to extract biologically relevant patterns; 4) Identification, on the help of visual representations, of interesting sequential rules and generation of particular gene-gene interaction hypotheses; and 5) Exploration of most interesting hypotheses (either by accessing to the list of GO annotation terms matches or by performing intensive literature search). Then, they participated in the writing of the results and discussion sections of the present manuscript.

Full details for these evaluation guidelines committed by field-experts during the interpretation of model results have been addressed in the method subsection: “Problem and Datasets Description: Long-term Interventions in Obesity”

**(Pages 25 and 26)** Lines 698-719 **Method** Section.

Moreover, clarification of this study strategy has been also included in the introduction section:

**(Pages 10 and 11)** Lines 279-288 **Introduction** Section.

**RV:**

3. The following sentences are in the results section, and I believe they are intended to be interpreted as results from this study: “In general, extracted rules presented good values for all computed quality measures, which indicates a good performance of the algorithm during the gene association mining process.” and “Of note, top sequential rules also presented good rates in the new proposed biological quality measures.” Describing values or rates as good is not informative unless the reader has a benchmark to compare to, and even then it should be avoided. As such, I do not know how to interpret much of the results in the Results section.

**AA:**

Thank you for your comment. CMRules counts on several quality assessment metrics that, based on frequency data, allow an easy identification of the robustness of each extracted rule by simply using a reference range of values. In order to make clearer the technical concepts in which our sequential rule mining method is based on, we have extended the available information in our method subsection “**Preliminary: Association Rules and Sequential Rules**”. In this section of the manuscript, we have now detailed how up to five quality metrics and a framework for rule selection have been developed in order to guide the generation and evaluation of model results. Please, read:

**(Pages 11 and 15)** Lines 299-411 **Method** Section.

In any case, we have rewritten the result sentences highlighted by the reviewer, now making explicit mention to the reference range of values of each metric and avoiding the use of the adjective “good” whenever possible:

**(Pages 27 and 28)** Lines 746-770 **Result** Section.

**RV:**

4. While the authors indeed mention that “Although extracted sequential rules may seem to indicate causal relations among genes, it is important to note that, in the majority of the cases, they just represent indirect relationships between items,” I would have liked them to dive more into what it means for two nodes to have a directed connection in their reconstructed networks. The authors say that the connections “might be illustrating how different biological processes take place in AT one after the other, and how there may exist some functional connections between them,” but this still does not address the basic question of what a network edge means.

**AA:**

Thank you very much for this observation. We decided to include these commentaries with the aim of illustrating the importance or the need for our introduced functional validation framework. We are sorry that they could be misunderstood and now we have removed the sentences from the discussion section.

Our employed method is intended to discover causal relationships between items. Nevertheless, since the ML process is guided or based on the use of quality metrics that are computed with frequency data, it could happen that some rules without biological sense be generated sometimes.

Within this context, the main utility of our presented biological quality measures is to distinguish between true potential causal gene-gene interactions (**rules 1 and 4**) and those representing spurious biostatistical fluke (**rules 2 and 3**).

In the presented figure, while all extracted rules present acceptable and identical quality metrics (support=90% and confidence=85%), only the **rule 1** presents a good BP measure value (remember that the range of values available for the BP measure was from 5 to 1, being the values near to 1 the ones corresponding to a higher number of GO matches between LHS and RHS genes). On the other hand, it is only the **rule 4** the one presenting a good value for the TF measure (whose range of values was from 0 to 3, being 3 the maximal score for indicating a true TF-target gene relationship).

Particularly, this figure represents how although the statistical quality metrics by rule may be indicating a robust association between items, not always a gene in the LHS will be the cause of the change in the expression of a RHS gene from a biological point of view. Indeed, in some cases, rules could be just referring to a range of parallel phenomena that occur simultaneously in a database at the gene level because of simply co-expression. Contrarily, in other cases, the method could be effectively representing true causal relationships between genes (e.g. **rules 1 and 4** from the figure). Precisely to discern between these spurious associations and those true phenomena of interaction, is why we propose the functional validation of our results and why some of the biological quality measures presented, such as the TF measure, become especially relevant.

These kind of phenomena have been also highlighted in our work by the absence of correlation between **some** biological quality measures and traditional quality metrics (Fig 4), which reinforces the fact that although traditional metrics may indicate that some rules are robust from the frequentist point of view, the biological information is not always what it could be expected.

All these thoughts and the presented figure have been included in the manuscript as part of the main text:

Figure 2.

**(Page 22)** Lines 609-620 **Method** Section.

**(Pages 27 and 28)** Lines 762-770 **Results** Section.

**Following reviewer recommendations** and given the fact that **a reader could still be wondering what does it mean for two genes to be connected by a sequential rule in our experiment**, we next **deepen into the concept of sequential association rule**, interconnecting it with results derived from our cases of study. With next examples, we hope to clarify the types of biological interactions that our pipeline could detect in a temporal GE dataset.

Sometimes, interactions could be indicating how the increase in the amount of a transcription factor causes a subsequent increase or decrease in the expression of a target gene, while other times they could be suggesting how two distinct genes (participating in a same metabolic route) increase their expression consecutively after an intervention. In the latter case, for example, the interaction would be illustrating how the activation of certain biological pathway is maintained over time in response to an intervention, after a first trigger event. Additionally, in other cases, when it is the same gene the one that occupies both LHS and RHS positions, rules could be suggesting negative or positive feedback phenomena (which could serve as explanation for fast transient dynamic changes or the maintaining in time of expression levels of certain genes in long-term interventions).

Interestingly, all introduced types of relationships have been reported in our tested datasets and assert with the two core ideas of XAI:

•	Explainable models, while maintaining a high level of learning performance (e.g. support, confidence, conviction, CF, lift).

•	Enabling human users to understand, appropriately trust, and effectively manage the emerging generation of artificially intelligent methods.

This information has also been included in the text:

**(Pages 33 and 32)** Lines 944-962 **Discussion** Section.

As we have previously mentioned, all introduced types of interactions discusses here are examples of some of the behaviors encountered in our case of study results:

- Example of relation between a transcription factor and its target (extracted from Lines 848-855 Results Section)

***“****From both intervention groups, several similar rules were identified sharing the same TF gene (Notch3) as an LHS item. In these rules, the gene Notch3 emerged as a TF factor whose downregulation (provoked by the WL intervention) elicited later secondary changes in the expression of other genes during the WS period. Since each group was mined independently during the knowledge-extraction process, the fact of finding similar rules from each diet speaks well of the performance and the validation ability of our method. Notch3 is mammalian transmembrane protein that bind membrane-bound ligands expressed by adjacent cells in human tissues. By triggering intracellular proteolytic cleavages and through the release of active intracellular domains of Notch (NICD), Notch3 controls the expression of a wide range of target genes participating in different obesity-related processes such as differentiation, proliferation, angiogenesis and apoptosis. Interestingly, several Notch3 target sequences have been identified within and near the genomic sequences of a few of its RHS genes (such is the case of Nmt2 and Clmn). In these cases, sequential rules illustrate how a downregulation of the Notch3 is followed by a downregulation and upregulation (respectively) of mentioned RHS genes. Despite these interesting results, it is important to clarify that the identification of sequential rules including a TF as LHS does not necessarily imply a causal relationship between the TF and its reported RHS gene. In these cases, functional in vitro studies should be performed for validating proposed interactions. From the LCD group it is also remarkable a rule with the Notch3 as LHS and the Egfl6 gene as RHS (Fig 2). Although no target sites for Notch3 have been identified within the genetic sequence of Egfl6, a special functional connection has been evidenced between both genes in the context of obesity and angiogenesis. In general, despite only a few previous evidences support an implication of the Notch3 gene in obesity molecular pathways, the findings presented in this paper seem to point this TF as an important element for the proper regulation of AT cellular responses to WL.****”***

*“In order to validate and contrast the insights derived from the discovery dataset, we accessed additional temporal GED from three WL interventions performed in independent cohorts (GSE70529, GSE35411 and GSE103766). As a result, we found gene expression changes in 11 loci acting as trigger mechanisms (LHS items) concurrently in sequential rules extracted from different datasets (these were C6=Up-regulation, Hnrnpa1=Up-regulation, Srsf7=Up-regulation, Gsap=Up-regulation, Sncg=Downregulation, Notch3=Downregulation, Srpx=Up-regulation, Itpka1=Downregulation, slc-transporters=Downregulation, Tmem-proteins=Downregulation and Znf-proteins=Up-regulation). Interestingly, these validated trigger loci included TFs, splicing factors, mRNA processing molecules and cell surface transporters with a great implication in the control of the global gene expression cell profiles.”*

- Example showing the maintaining in time of a particular biological or metabolic pathway activation after an intervention (extracted from Lines 800-807 Results Section)

*“For investigating the sequential rules discovered in the VLCD group (Fig 4), the use of other biological quality measures instead of TF (such as BP and MF) allowed experts to identify several interesting patterns. On the one hand, it highlights a sequential rule involving the loci Fasn (Fatty Acid Synthase) and the Gpam (Glycerol-3-Phosphate Acyltransferase 1, Mitochondrial); both of them genes coding for enzymes with a central role in the process of lipogenesis. The sequential rule between these genes was easily identified from its color intensity in the circular plot and may suggest a special relevance of the lipogenesis process as part of the responses of obese AT after a strong caloric restriction.”*

- Example of negative or positive feedback phenomena (extracted from Lines 879-886 Results Section)

*“Particularly, we observed how although certain genes experimented a downregulation of their gene expression in response to WL, these genes returned to their original gene expression status as soon as a normal-calorie diet was restored (exhibiting some kind of negative and positive feedback loop regulations of their own expression, which could be the explanation of fast transient dynamic changes or the maintaining in time of their expression levels). Altogether, these validated patterns might represent the sequence of genetic changes that occur in AT during a long-term weight loss intervention. Indeed, some of the identified loci have already been drawn as key genes or targets for the management of many complex diseases.”*

In all previous examples, it is clear how our method could serve as a link between high throughput omics analytics approaches and functional validation studies.

**RV:**

**III. Minor comments and non-urgent requests**

1. I would earnestly like to offer substantive, line-by-line feedback on this article, but the format often made it challenging. In the future, small things like page numbers and line numbers can go a long way towards helping reviewers offer substantive feedback.

- I strongly recommend referring to the PLoS Computational Biology manuscript formatting page hyperlinked here prior to submitting this article.
- If the authors write their papers using LaTeX, PLoS Computational Biology also has a publicly-available template, hyperlinked here.

2. Table 1 is very disorganized and makes the descriptions inside it hard to follow.
3. There are so many acronyms and abbreviations, and it is often not clear if they refer to the authors’ own methodology or if they are common in the machine learning literature.

**AA:**

1. Thank you for your suggestion, we have adapted our Microsoft word document to the mentioned format.
2. We have improved the format of table 1 making it clearer.
3. Following reviewer’s recommendation, we have avoided the use of unnecessary abbreviations through by the manuscript, considerably reducing their number.

**RV:**

4. While the graphical abstract is visually appealing, I struggle to interpret most of it. The icons are sleak, but I think that simpler figure that focuses on the specific contributions in this work is much more compelling.

**AA:**

Thank you for your comment. The confection of the proposed graphical abstract was ordered to a professional graphic design study as part of an economic outlay committed by our group past months. Now, the labor relationship with the graphic design study responsible for that figure has ended and we are not in disposition to order new changes regarding the work. Moreover, unfortunately, we have not enough graphic design knowledge in order to edit the figure by ourselves.

In spite of it, if the reviewer consider it appropriate we could made an effort in order to generate a simpler new version.

Anyway, since the *PlosCompBiol Journal* does not require the submission of such type of figure, our presented graphical abstract could be considered as an extra work and we could discard it without any problem (although we would prefer not to do so). We let at the reviewer and editor’s discretion how to proceed in this regard.

Response to Reviewer 2 Comments

**RV: Reviewer**

**AA: Author Answers**

**RV:**

This paper tackles the problem of explainable artificial intelligence for omics data. As such, the topic is currently very interesting as most existing techniques are black-boxes, hence, they are not interpretable for clinicians, biologist, etc.

The authors use a novel rule-based strategy that includes preprocessing, knowledge extraction and biological validation to tackle the problem. They have run experiment on in-vivo GED in 57 subjects with obesity.

Overall, the paper is very well-written, it is easy to follow and the contribution is clearly relevant for a great audience. In addition to that, I am really happy to see that the authors are sharing all the source code generated on Github.

**AA:**

We would like to thank the reviewer for taking a time to revise our manuscript and for highlighting such positive comments. We have accomplished proposed his/her minor suggestions in order to improve as much as possible the final paper quality.

**RV:**

I only have some minor suggestions:

- As the case study is very much focused on obesity, perhaps the title should also reflect that..as this pipeline might not always work in GED.

**AA:**

Following the reviewer’s recommendation, **we have changed the title** of our proposal and have included a sentence referring to the field of study in which we validate our approach. We thought it would be appropriate since the application of our methodology to the case of study is a crucial step for evidencing its interpretability and for validating its performance. Moreover, we think that some of the case of study results presented in our paper could be interesting to a broader biologist audience, and thus they should be reflected in the title.

“eXplainable Artificial Intelligence (XAI) for the identification of biologically relevant gene expression networks patterns in longitudinal human studies, Insights from obesity research.”

Anyway, we would like to point that the work presented here is a **methodology** intended to work with **any type of GED** (not only obesity datasets), whenever they come from a long-term intervention in humans (with at least three temporal points analyzed). Indeed, as input files, our pipeline receives raw fluorescence intensity signals (one .cel file per subject and time point), and perform a transformation into the form of an N x M matrix of gene expression values, where the N rows correspond to subjects under study and the M columns correspond to evaluated gene probes. From then on, all steps of our method are take place one after another.

Since we have noticed that in our **Methods** section there was not a clear separation between the parts corresponding to the pipeline description and the parts corresponding to the description of the application of our method to the example datasets, we have improved the **description of the technical parts of the methodology**.

For that purpose, the whole **method section has been rewritten**, illustrating how our method could be applied to **a hypothetical gene expression dataset** (not focused in the obesity field):

**(Pages 11-23)** Lines 289-640 **Method** Section.

Otherwise, the technical particularities regarding the **application of the pipeline to example datasets** have been separately gathered in:

**(Pages 23-26)** Lines 642-719 **Method** Section.

**RV:**

- Data preprocessing seems to be focused only on discretisation, which is definitely key. Just wondering if the authors could discuss other forms of data preprocessing. For example, feature reduction (selection) may be very interesting with this kind of data.

**AA:**

Thank you for the comment.

Beside the data discretization, **feature selection has also been implemented** in our pipeline. Particularly, this feature selection process consisted on **selecting potentially interesting genes that were affected by the intervention** conducted.

The motivation for this step was the high number of probes/genes available in the Affymetrix platforms analyzed (often around 33.000), which may induce an exponential growth of the search space. Therefore, the knowledge-extraction process (independently of the ML method used) could become a difficult and complex task exceeding the processing capability of conventional systems. In order to solve this problem, prior to knowledge-extraction, our methodology includes a processing step in which the number of probes is reduced according to the differentially expressed (DE) genes by time interval and experimental condition. Given a longitudinal GED experiment, our method identifies DE probes by assessing the changes in gene expression during each period of intervention. A probe will be selected for downstream analyses when its Bonferroni-adjusted P-value is < 0.05 and the associated Log_2_(FoldChange) (which is also known as the signal log ratio) is >= 1 or <= -1 in a paired t-test with Bayesian correction.

All this information has been included in the appropriate method section of our manuscript:

**(Page 16)** Lines 425-435 **Method** Section.

**RV:**

- It is clear that the results are interpretable, and the figures and table reported are proof of that. However, I would like to see a bit of additional discussion about this; in particular, would this approach always be interpretable? what are the caveats to use this approach? Maybe good to discuss potential future work from here.

**AA:**

We thank the reviewer for the comment.

We humbly think that our approach will be interpretable whenever it is accompanied of its indispensable functional validation step.

Results from our SRM method are understandable by itself (in the form of association rules; X -> (time delay) Y). Nevertheless, without their biological validation, we could not ever be sure if reported rules represent true gene-gene interactions or just biostatistical fluke, which could happen since the ML process is based on the use of quality metrics that are computed with frequency data. Thus, the main source of interpretability in our approach is achieved through the use of biological quality measures by rule and through the visual interpretation of results.

We have illustrated the announced problematic with the next figure. Within this context, the main utility of our presented biological quality measures is to increase model interpretability by distinguishing between true potential causal gene-gene interactions (**rules 1 and 4**) and those representing spurious biostatistical fluke (**rules 2 and 3**).

In the figure, while all extracted rules present acceptable and identical quality metrics (support=90% and confidence=85%), only the **rule 1** presents a good BP measure value (remember that the range of values available for the BP measure was from 5 to 1, being the values near to 1 the ones corresponding to a higher number of GO matches between LHS and RHS genes). On the other hand, it is only the **rule 4** the one presenting a good value for the TF measure (whose range of values was from 0 to 3, being 3 the maximal score for indicating a true TF-target gene relationship).

All these thoughts and the presented figure have been included in the manuscript as part of the main text:

Figure 2.

**(Page 22)** Lines 609-620 **Method** Section.

**(Pages 27 and 28)** Lines 762-770 **Results** Section.

More discussion on the interpretability of our approach has also been amended in the discussion section:

**(Pages 33 and 32)** Lines 944-962 **Discussion** Section.

Regarding **future lines of research**, next topics have been included in the discussion section:

- The application of our pipeline to RNAseq datasets.
- Improving of the computing process for biological quality measurements based on go terms.
- The use of alternative ways of discretizing data and performing feature selection.

**(Pages 36-37)** Lines 1011-1039 **Discussion** Section.

Response to Reviewer 3 Comments

**RV: Reviewer**

**AA: Author Answers**

**RV:**

Naming the step where rules are overlaid in biological databases “Biological validation” may be misleading. What the authors refer in this step will be more in the framework of Functional validation. Please rephrase accordingly in the main text, supplementary material and code.

**AA:**

We would like to thank the reviewer for taking a time to revise our manuscript.

Regarding this first comment, we agree with the reviewer in the fact that “biological validation” could not be the most appropriate term for that procedure and thus we have changed it by “functional validation” through all parts of the manuscript where it appeared.

**RV:**

- Rules for scoring:

Please explain how the authors decided on the scoring system for each category. Are they purely heuristic? Have the author evaluated the effects of varying the assigned values?

For matching terms of LHS and RHS, are the inherent term ontologies in the databases considered? E.g. would a child-ancestor pair be considered a hit? Please explain why yes/no.

**AA:**

Thank you very much for this observation. We agree with the reviewer in the fact that the computing process by which presented biological quality measures by rule (based on GO and KEGG terms) are estimated is an interesting point to discuss.

Just with the aim to remember, the computation process for these four quality measures in our work is displayed in Fig 1. The choice of that particular procedure was argued in the fact that we needed an evaluation method, **based on categories**, with the ability to discern the quality of a rule regardless of the items conforming it. If this were not the case, the rules with the highest number of antecedent/consequent elements would always have a higher score by the simple chance of coinciding in GO terms due to the greater number of genes composing them. On the contrary, with our heuristic approach, the score is adjusted by the number of items (from the total elements that constitute the rule) that share a specific GO term. Thanks to that, the rules in which all its elements share the same GO term (**though only one**) will be identified as more robust than others, which although perhaps share a **greater** number of GO terms among all its antecedent elements, do not share **any** GO between LHS and RHS items.

Although our approach is interesting and we have found it useful to discern the top rules of our sets, alternative tools have been developed to evaluate the biological similarity of two genes based not only on the identical GO terms that they share, but also on the rest of GO terms that are annotated (not identical) (Please, s*ee the work of* [*Pesquita et al. (2009)*](https://journals.plos.org/ploscompbiol/article?id=10.1371/journal.pcbi.1000443) *published in the Plos Comp Biol)*. Some of the most interesting approaches in this regard are based on counting the connections between two different GO nodes according to the gene ontology database network and correcting the derived result by how common these terms are in terms of whole human genome annotation. With this approach, some rules that our method currently identifies as not so interesting rules from the GO perspective, could then be highlighted as rules of moderate interest according to the non-identical GO terms of the genes that make them up. In any case, the best option would be a combination of our heuristic method alongside this more sophisticated approaches. In the future, a combined approach like this could be of great interest to improve the functional validation of our method and will be taken into consideration for the continuation of this work. Besides this modification, other future approaches could also consist of performing the visual representation of the rules with ontology terms representing nodes instead of genes. This would allow us to visualize networks in terms of functionality and understand how cellular functions follow each other in human tissues after long-term interventions. In this case, the difficulty would be to identify which GO terms are the most characteristic for each gene in order to represent them within the network. Once achieved, the way in which the nodes of the network are connected could be different to our current representations and thereby reveal novel information extracted by the method that is not observed with our current approach.

In any case, the biological quality measurements based on GO terms are only three of the **five functional validation measures** that we have introduced in our analysis. Thus, they are complemented, together with the other measures (TF and SP), so that the user is able to discern possible top rules within the network. As they have shown in the datasets under study, all of them have served to generate biologically relevant particular hypotheses of gene-gene interactions for researchers. Particular hypotheses that could then be explored in more detail either by accessing the list of GO annotation terms of each item, continuing the process of literature search, or at the experimental level in wet-lab.

In order to improve the discussion of these issues in our manuscript, new parts have been added in next sections:

**(Pages 21-22)** Lines 590-599 **Method** Section.

**(Pages 36-37)** Lines 1026-1039 **Discussion** Section.

**RV:**

- Discretization is a key aspect in the presented methodology and merits deeper explanations. In the main text is stated:

“- For probes showing a positive signal log ratio:

If log2(FoldChange)ikj > log2(FoldChange)ikJ Then assign the label ‘Upregulation’.

Otherwise type ‘No change’

- For probes showing negative signal log ratio:

If log2(FoldChange)ikj < log2(FoldChange)ikJ Then assign the label ‘Downregulation’.

Otherwise type ‘No change’

Where the term log2(FoldChange)ikj refers to the change in the gene expression for the i probe, in the k time interval and the j subject, and the term log2(FoldChange)ikJ otherwise refers to the mean signal log ratio for that particular ik probe in all subjects from the group under study (our designated data scope).”

a) For log2(FoldChange)ikJ I believe the authors are referring to mean signal log ratio for a particular probe i at a given time point k in all subjects from the group under study J. Please clarify.

b) Probes need to be defined in the sentence “probes showing a positive/negative signal log ratio”. I believe they refer to a probe ikj (probe, timepoint, subject)

c) If I’m correct in b) I can think of several cases where down/up regulation events are missed out. For example for a given sign for the log ratio where the group has inverted signed. Also when the log ratio is positive but significantly lower than the group (downregulated). The opposite case, when the log ratio is negative but significantly higher than the group (upregulated). Please clarify.

**AA:**

Thank you for arising this point.

Data discretization was included in our pipeline as the second data processing step.

The main motivation behind the application of GED discretization was allowing the use of ML algorithms, such as SRM, that requires discrete data as an input for the inference of biological knowledge. Nevertheless, there are many other advantages that arised from data discretization in genetics; 1) Discrete states favor the inference of qualitative models, which are of special importance in terms of model explicability. 2) Another advantage emerging from GED discretization was the homogenization of different datasets in terms of interpretability. 3) Finally, the learning process from discrete data is more efficient and effective (requiring a reduced amount of data and yielding more compact and shorter results). Therefore, this step not only allows the inference of large-size models with a higher speed of analysis but also facilitate a significant portion of the biological and technical noise presented in the raw data to be absorbed, which may indirectly lead to a better model accuracy.

Regarding **a)** and **b)** comments:

The reviewer was right in their suppositions about the meaning of these sentences. Now, we have tried to clarify them following reviewer recommendations:

**(Page 17)** Lines 465-475 **Method** Section.

Regarding the **c)** comment:

We have given a deeper explanation of the choice of our data discretization approach, comparing it against similar approaches in literature. The discretization approach adopted in this work can be viewed as an extension of two previously described in vitro temporal methods that have been successfully applied for the reconstruction of gene regulatory networks. The main motivation behind its choice is the fact that, when facing temporal GED, discretization methods based on transitions between time-points have been shown to obtain better results than those using absolute values. Moreover, in our case, the use of a standardized measure of gene expression change (such is the Signal Log Ratio (SLR)) is a more sophisticated approach than the employment of simple differences between values. For example, the use of logs in the analysis eliminates difficulties caused by one very high data point in the set masking information from lower valued data points. On the other hand, base 2 is further used as the log scale, therefore a SLR of 1 represents a two-fold increase in abundance of an mRNA and a value of –1 represents a two-fold reduction in transcript expression. Finally, the use of the mean SLR (log2(FoldChange)ikJ) as the threshold for discretization allowed us to exclusively focus on general change trends in the dataset, which most likely will be consequence of the intervention conducted in the cohort and not particular gene expression changes due to individual’s idiosyncrasy. Mapping data from a vast spectrum of numeric gene expression values to a reduced subset of three discrete states, this type of discretization could further be viewed as a secondary data reduction technique in favor of algorithm efficiency and eXplainability. This discussion has been also included in the paper. Please see:

**(Pages 16-18)** Lines 436-492 **Method** Section.

**RV:**

- Please explain in Supp Fig 1. what 1,2, or 0 means in the Discretization step.

**AA:**

This issue has been clarified in the discretization method subsection

**(Page 17)** Lines 459-462 **Method** Section.

and also in the caption of the Supp Fig 1

**(Page 45)** Lines 1268-1274 **Table and Figure Legends** Section.

**RV:**

- As NGS techniques such RNA-seq are now standard with a variety of applications, the paper will benefit by mentioning if or how the proposed methodology can be applied.

**AA:**

We thank the reviewer for this suggestion. We agree with him/her in the fact that NGS techniques present and advance both in the technical and practical dimensions from microarrays experiments. For this reason, we have specified what is needed to apply our pipeline to such kind of data.

For this to be done, it would be enough by with counting on an *N x M* gene expression matrix of normalized reads (as detailed in the method subsection). In future works, it would be of great interest testing our approach with RNAseq expression datasets given the stronger reliability of these data in term of the technical robustness of sequencing platforms. Approaches like this would greatly expand our knowledge for complex biological processes, with a special interest for long-term intervention experiments (such as clinical trials), in which gene regulatory mechanisms could reveal new drug targets.

**RV:**

I particularly enjoy that the source code is made available and shared. I think that some more work is needed in the usability of the methodology in order to allow the scientific community to use this novel approach:

- As much as possible avoid hard-coded filenames in all steps. One can use Rscript to run R with command line arguments.

- As much as possible provide a simplified execution: Pipeline steps which are feeding directly from the output of the previous step or can run sequentially. For example step 1, 2, and 3.

- For step 3 avoid asking the user to manually prepare the format. The instructions can be rather easily done programatically.

- Avoid as much as possible asking for having superuser rights (step 4). This is very limiting if working in shared computing facilities. If it's a must, then a virtual environment may help.

- Minor: In the source code "required _codes": rename to src or just place them in the root folder.

**AA:**

Thank you very much for highlighting this positive comment.

Following reviewer’s recommendation, we have implemented almost all changes in the source code (except converting R scripts into a function with imputable arguments). Consequently, a new version of the software has been uploaded to the GitHub repository.

Particularly, pipeline steps which feed directly from the output of the previous step have been merged into one single script (step 1 – pre-processing). Step 3 has been implemented programmatically and the directory “required_codes” has been renamed to “src”.

Regarding the ask for superuser rights in the step 4, it is not a mandatory condition but a recommendation for avoiding problems in some operative systems such as Linux.

We hope the whole set of codes is more understandable and easy to use now.
